# Genome-Wide Annotation and Comparative Analysis of Cytochrome P450 Monooxygenases in Basidiomycete Biotrophic Plant Pathogens

**DOI:** 10.1371/journal.pone.0142100

**Published:** 2015-11-04

**Authors:** Lehlohonolo Benedict Qhanya, Godfrey Matowane, Wanping Chen, Yuxin Sun, Elizabeth Mpholoseng Letsimo, Mohammad Parvez, Jae-Hyuk Yu, Samson Sitheni Mashele, Khajamohiddin Syed

**Affiliations:** 1 Unit for Drug Discovery Research, Department of Health Sciences, Faculty of Health and Environmental Sciences, Central University of Technology, Bloemfontein 9300, Free State, South Africa; 2 College of Food Science and Technology, Huazhong Agricultural University, Wuhan, Hubei Province, China; 3 Department of Bacteriology, University of Wisconsin-Madison, 3155 MSB, 1550 Linden Drive, Madison, WI, 53706, United States of America; Kansas State University, UNITED STATES

## Abstract

Fungi are an exceptional source of diverse and novel cytochrome P450 monooxygenases (P450s), heme-thiolate proteins, with catalytic versatility. Agaricomycotina saprophytes have yielded most of the available information on basidiomycete P450s. This resulted in observing similar P450 family types in basidiomycetes with few differences in P450 families among Agaricomycotina saprophytes. The present study demonstrated the presence of unique P450 family patterns in basidiomycete biotrophic plant pathogens that could possibly have originated from the adaptation of these species to different ecological niches (host influence). Systematic analysis of P450s in basidiomycete biotrophic plant pathogens belonging to three different orders, Agaricomycotina (*Armillaria mellea*), Pucciniomycotina (*Melampsora laricis-populina*, *M*. *lini*, *Mixia osmundae* and *Puccinia graminis*) and Ustilaginomycotina (*Ustilago maydis*, *Sporisorium reilianum* and *Tilletiaria anomala*), revealed the presence of numerous putative P450s ranging from 267 (*A*. *mellea)* to 14 (*M*. *osmundae*). Analysis of P450 families revealed the presence of 41 new P450 families and 27 new P450 subfamilies in these biotrophic plant pathogens. Order-level comparison of P450 families between biotrophic plant pathogens revealed the presence of unique P450 family patterns in these organisms, possibly reflecting the characteristics of their order. Further comparison of P450 families with basidiomycete non-pathogens confirmed that biotrophic plant pathogens harbour the unique P450 families in their genomes. The CYP63, CYP5037, CYP5136, CYP5137 and CYP5341 P450 families were expanded in *A*. *mellea* when compared to other Agaricomycotina saprophytes and the CYP5221 and CYP5233 P450 families in *P*. *graminis* and *M*. *laricis-populina*. The present study revealed that expansion of these P450 families is due to paralogous evolution of member P450s. The presence of unique P450 families in these organisms serves as evidence of how a host/ecological niche can influence shaping the P450 content of an organism. The present study initiates our understanding of P450 family patterns in basidiomycete biotrophic plant pathogens.

## Introduction

For the last five decades cytochrome P450 monooxygenases (P450s/CYPs), heme-thiolate proteins, have been in the spotlight [1] because of their catalytic versatility [2] and potential biotechnological value [3, 4]. These enzymes can be found in all living organisms belonging to different biological kingdoms [5] and are known to play a key role in organisms’ primary and secondary metabolism, including degradation of xenobiotic compounds [1]. Because of their stereo- and regio-specific oxidation of substrates, these enzymes have become critical in organisms’ survival [6–9]. Among biological kingdoms, the fungal kingdom and species in them present a large amount of information on different aspects of P450s. The aspects include P450 family diversity [10], catalytic versatility [11–13], P450 family enrichment [13], thermostable P450s [14], P450s as drug-target [4, 15] and provision of biotechnologically valuable P450s [4].

Analysis of P450s in fungi revealed the highest P450 family diversity in ascomycetes compared to basidiomycetes [16]. Species belonging to Ascomycota and Basidiomycota show different P450 family types in their genomes [17]. Basidiomycetes are one of the unique fungi capable of complete degradation of plant material including the most recalcitrant plant cell wall component, lignin [18]. The recent explosion in genome sequencing of basidiomycetes helped researchers to understand the P450 patterns, their distribution and evolution in these organisms [13, 14, 16, 17, 19–22]. However, the available knowledge on basidiomycete P450s mostly came from wood-degrading species belonging to the order Agaricomycotina. Analysis of P450s across the Agaricomycotina species, such as *Phanerochaete chrysosporium* [23, 24], *P*. *carnosa* [19], *Postia placenta* [25, 26], *Ganoderma* sp. [20]. *G*. *lucidum* [27], *Agaricus bisporus* [28, 23], *Ceriporiopsis (Gelatoporia) subvermispora* [29], *Serpula lacrymans* [13], *Bjerkandera adusta* and *Phlebia brevispora* [20], revealed almost the same P450 family patterns, despite the presence of a few different P450 families in these species. Furthermore, recent study revealed that certain P450 families in these organisms are enriched *via* paralogous evolution of member P450s to help the organism adapt to different ecological niches, such as colonization on plant material [13].

Apart from the dead wood degrading basidiomycetes described above, efforts were made regarding genome sequencing of basidiomycete biotrophic plant pathogens to understand the virulence factors responsible for pathogenesis [30–36]. The basidiomycete biotrophic plant pathogen genomes sequenced include the biotrophic ubiquitous parasite of maize and a model fungus for the study of microbe-plant pathogen interactions, *Ustilago maydis* [30], another maize biotrophic pathogen, *Sporisorium reilianum* [35], an intracellular rice pathogen, *Tilletiaria anomala* [34], obligate biotrophic pathogens of crop plants, *Melampsora laricis-populina* [31] and *M*. *lini* [33], *Puccinia graminis f*. sp. *tritici* [31], an intracellular pathogen of ferns, *Mixia osmundae* [36] and the ubiquitous plant pathogen *Armillaria mellea* [32]. Contrary to the basidiomycete plant pathogens, their counterparts, ascomycete plant pathogen P450s, have been extensively characterized through profiling of P450s in these organisms and also functional analysis of some P450s [16,17,21,22].

To date, systematic and comparative analysis of P450s in basidiomycete plant pathogens, especially biotrophs, has not been carried out. Previous studies on comparative analysis of P450s in fungi [16, 17, 21, 22] focused on different aspects of fungal P450s. A thorough analysis of P450s in the basidiomycete biotrophic plant pathogens has not been reported. In this study, we performed genome-wide identification, annotation and evolutionary analysis of P450s in basidiomycete plant pathogens, especially biotrophs belonging to three different orders: Agaricomycotina (*A*. *mellea*), Pucciniomycotina (*M*. *laricis-populina*, *M*. *lini*, *M*. *osmundae* and *P*. *graminis*) and Ustilaginomycotina (*U*. *maydis*, *S*. *reilianum* and *T*. *anomala*). Furthermore, we performed comparative analysis of biotrophic plant pathogen P450s with non-pathogen fungi and focused on the analysis of P450 patterns between different orders and P450 family expansion, if any, in these biotrophic plant pathogens.

## Materials and Methods

### Species selection and P450 mining

Basidiomycete biotrophic plant pathogens and non-pathogens belonging to three different orders were selected for analysis of P450s (Table 1). These species’ genome sequencing data have been published and are available for public use (Table 1). P450 mining in these species was carried out following the methodology that has been described meticulously in the literature [13, 14, 16, 19–21, 26, 37, 38]. Briefly, putative proteomes of each species were downloaded from the respective species’ databases listed in Table 1. The putative proteome was subjected to the NCBI Batch Web-search tool [37] to separate proteins into different functional categories. The proteins that were grouped under the P450 superfamily were selected and checked for the presence of two P450 signature motifs, FXXGXRXCXG (also known as CXG) in the heme-binding domain and the EXXR motif in the K-helix [39–41]. Identification of P450s in organisms depend solely on identification of these two P450 signature motifs and this method is well documented in the literature, particularly any P450 showing one of the motifs considered pseudo-P450 [13,14,16, 19–21, 26, 37, 38]. The sequences that showed both motifs were selected for naming. The length of selected P450s is >400 amino acids, indicating that these P450s are full-length. For this reason assigning the family and subfamily can be done without any errors. The P450s that showed one of the motifs represent pseudo-P450s, hence they were not annotated. The P450s of *Malassezia globosa* that have been annotated and are available for public use at Cytochrome P450 Homepage [42] are used in this study.

**Table 1:** Information on databases used to download the whole proteomes of basidiomycete biotrophic plant pathogens and non-pathogens. All databases were located at the MycoCosm portal of the Joint Genome Institute (JGI), United States Department of Energy (US-DOE) [43].

Species name	Database	Reference
**Order: Agaricomycotina**		
*Armillaria mellea*	http://genome.jgi.doe.gov/Armme1_1/Armme1_1.home.html	[32]
**Order: Pucciniomycotina**		
*Melampsora laricis-populina*	http://genome.jgi-psf.org/Mellp1/Mellp1.home.html	[31]
*Melampsora lini* CH5	http://genome.jgi-psf.org/Melli1/Melli1.home.html	[33]
*Mixia osmundae* IAM 14324	http://genome.jgi-psf.org/Mixos1/Mixos1.home.html	[34]
*Puccinia graminis*	http://genome.jgi-psf.org/Pucgr1/Pucgr1.home.html	[31]
*Rhodosporidium toruloides* NP11	http://genome.jgi-psf.org/Rhoto1/Rhoto1.home.html	[44]
**Order: Ustilaginomycotina**		
*Ustilago maydis*	http://genome.jgi.doe.gov/Ustma1/Ustma1.home.html	[30]
*Sporisorium reilianum* SRZ2	http://genome.jgi-psf.org/Spore1/Spore1.home.html	[35]
*Tilletiaria anomala* UBC 951	http://genome.jgi-psf.org/Tilan2/Tilan2.home.html	[36]
*Malassezia globosa*	http://drnelson.uthsc.edu/CytochromeP450.html	[42]
*Malassezia sympodialis* ATCC 42132	http://genome.jgi-psf.org/Malsy1_1/Malsy1_1.home.html	[45]
*Pseudozyma antarctica* T-34	http://genome.jgi-psf.org/Psean1_1/Psean1_1.home.html	[46]
*Pseudozyma hubeiensis* SY62	http://genome.jgi-psf.org/Psehu1/Psehu1.home.html	[47]

### Assigning family and subfamilies

The above selected P450s were assigned to different P450 families and P450 subfamilies using the methodology that is described meticulously in the literature [13, 14, 16, 19–21, 26, 37, 38]. Briefly, the above selected P450s were blasted at the Cytochrome P450 Homepage [38] against all named fungal P450s and P450s of *Postia placenta*, *Bjerkandera adusta*, *Phlebia brevispora and Ganoderma* sp. [26, 20]. Homolog P450s (henceforth referred to as reference P450s) that showed the highest percentage identity to putative P450s were noted. P450s were grouped into families and subfamilies based on the International Cytochrome P450 Nomenclature criteria, i.e. P450s showing >40% identity were assigned to the same P450 family and P450s that showed >55% identity were grouped under the same P450 subfamily [48–50]. P450s that showed less than 40% identity to annotated P450s were assigned to new P450 families with the kind help of Prof David R Nelson, University of Tennessee Health Science Centre, Memphis, Tennessee, USA. Furthermore, alignment of P450s on the phylogenetic tree was taken into consideration while assigning the family and subfamily to the putative P450s. The P450s of *P*. *graminis*, *U*. *maydis* and *M*. *globosa* have been annotated and are available for public use [42]. In this case, protein IDs for P450s in these organisms were assigned from their respective genomic databases (Table 1) if the machine-annotated proteins available on the respective species’ genomic databases (Table 1) showed 100% identity to the annotated P450s on the Cytochrome P450 Homepage [42].

### Construction of P450 phylogenetic tree

The P450 phylogenetic tree was constructed following the methodology previously described [22]. Briefly, the annotated basidiomycete P450s were aligned by adjusting them to the P450 profile hidden Markov model (PF00067, the Pfam protein families database, http://pfam.xfam.org/) with the HMMER package 3.1 (http://hmmer.janelia.org/) [51,52]. Then, the phylogenetic trees based on the above alignments were inferred by FastTree version 2.1.4 using the maximum-likelihood method (http://www.microbesonline.org/fasttree/) [53]. The generated tree data were submitted to iTOL (http://itol.embl.de/upload.cgi) for making phylogenetic trees [54].

### Agaricomycotina saprophytes P450s

P450s belonging to the Agaricomycotina saprophytes *P*. *chrysosporium*, *P*. *carnosa*, *A*. *bisporus*, *Ganoderma* sp., *P*. *placenta*, *S*. *lacrymans*, *B*. *adusta* and *P*. *brevispora* were resourced from published literature [13, 19, 10, 16] and used for comparison with *A*. *mellea* P450s.

### Gene-structure and gene tandem-duplication analysis

Analysis of the gene structure for selected P450 family members was carried out following the methodology described elsewhere [13, 14]. Briefly, selected P450 family members’ intron-exon arrangements were analysed. The length of exons was noted as an indication of possible gene duplication, if P450s showed conservation in the size of exons. Genomic localization of member P450s was also carried out to assess the tandem arrangement of P450s. Localization of P450s on a scaffold/node and their DNA region from start to end were noted and used for identification of tandemly duplicated P450s.

## Results and Discussion

### Basidiomycete biotrophic plant pathogens P450ome

Genome-wide data mining for P450s in basidiomycete biotrophic plant pathogens revealed the presence of a large number of P450s in *A*. *mellea* (267) (Table 2 and S1 Table). In comparison to *A*. *mellea*, the other seven plant pathogens showed the lowest number of P450s in their genomes (Table 2 and S1 Table). Among the seven plant pathogens *M*. *laricis-populina* showed the highest number of P450s (27) in its genome, whereas *M*. *osmundae* showed the lowest number of P450s (14) in its genome. The number of P450s in other plant pathogens is highest compared to basidiomycetes, the human pathogen *Cryptococcus neoformans* and mycoparasite *Tremella mesenterica* that show eight P450s in their genome [42, 55].

**Table 2:** Genome-wide annotation and comparative analysis P450s in basidiomycete biotrophic plant pathogens.

Order	Species name	No. of P450s	No. of P450 families	No. of P450 subfamilies
Agaricomycotina	*A*. *mellea*	267	30	65
Pucciniomycotina	*M*. *laricis-populina*	27	14	16
	*M*. *lini*	22	13	17
	*M*. *osmundae*	14	14	14
	*P*. *graminis*	17	9	10
Ustilaginomycotina	*U*. *maydis*	23	19	22
	*S*. *reilianum*	16	15	16
	*T*. *anomala*	17	11	15

### New P450 families and subfamilies in basidiomycete biotrophic plant pathogens

Annotation of P450 families (assigning the P450 families and subfamilies to the putative P450s) revealed the presence of new P450 families in basidiomycete biotrophic pathogens. Based on the International Cytochrome P450 Nomenclature criteria [48–50], *A*. *mellea* P450s can be grouped into 30 P450 families and 65 P450 subfamilies (Table 2). Among the remaining seven biotrophic plant pathogens, *U*. *maydis* and *P*. *graminis* showed the highest and lowest number of P450 families and subfamilies in their genomes (Table 2). Some P450s of *A*. *mellea* contain one of the two P450 signature motifs, hence these P450s are regarded as pseudo-P450s and not annotated. Future availability of good genomic DNA sequence and better gene prediction methods will facilitate the annotation of these P450s. For the same reason a single P450 is omitted from the annotation for *M*. *laricis-populina* and *M*. *lini*. The number of P450 families and subfamilies in each species is listed in Table 2. A detailed analysis of reference P450s used for annotation of basidiomycete biotrophic pathogen P450s is given in S2 Table.

Analysis of P450 families revealed the presence of 41 new P450 families and 27 new P450 subfamilies in these biotrophic plant pathogens (Table 3). *U*. *maydis* and *M*. *lini* showed the highest (12) and lowest (1) number of new P450 families in their genomes. Among new subfamilies, *M*. *lini* showed the highest number of new P450 subfamilies in its genome (8) and a single new P450 family was observed in *M*. *laricis-populina* and *S*. *reilianum* (Table 3). Detailed analysis of the number and name of the new P450 families and subfamilies identified in each biotrophic plant pathogen is presented in Table 3.

**Table 3:** Information on new families and new subfamilies found in basidiomycete biotrophic plant pathogens.

	Total number		Name	
	New families	New subfa-milies	New families	New subfamilies
*A*. *mellea*	5	4	CYP5417, CYP5431, CYP5622, CYP5623, CYP6006	CYP5366B1, CYP5154F1, CYP5142M1, CYP5340D1
*M*. *laricis-populina*	4	1	CYP5395-CYP5398	CYP5139J1
*M*. *lini*	1	8	CYP5399	CYP5152NSF, CYP5221NSF1, CYP5230NSF, CYP5232NSF, CYP5233NSF, CYP52233NSF 1 & NSF2, CYP5396NSF
*M*. *osmundae*	8	3	CYP5662-CYP5669	CYP5139S1, CYP5141M1, CYP522E1
*P*. *graminis*	4	3	CYP5230-CYP5233	CYP5152B1, CYP5221B1, CYP5221C1
*U*. *maydis*	12	3	CYP5025-CYP5034, CYP5643, CYP5644	CYP53C1, CYP504C1, CYP504D1,
*S*. *reilianum*	3	1	CYP5032, CYP5636, CYP6007	CYP5640B1
*T*. *anomala*	4	4	CYP5026, CYP5367, CYP5639, CYP5641	CYP5028B1, CYP5031B1, CYP5031NSF, CYP5076D1

### Phylogenetic analysis of P450s and their clade features

**Fig 1:**
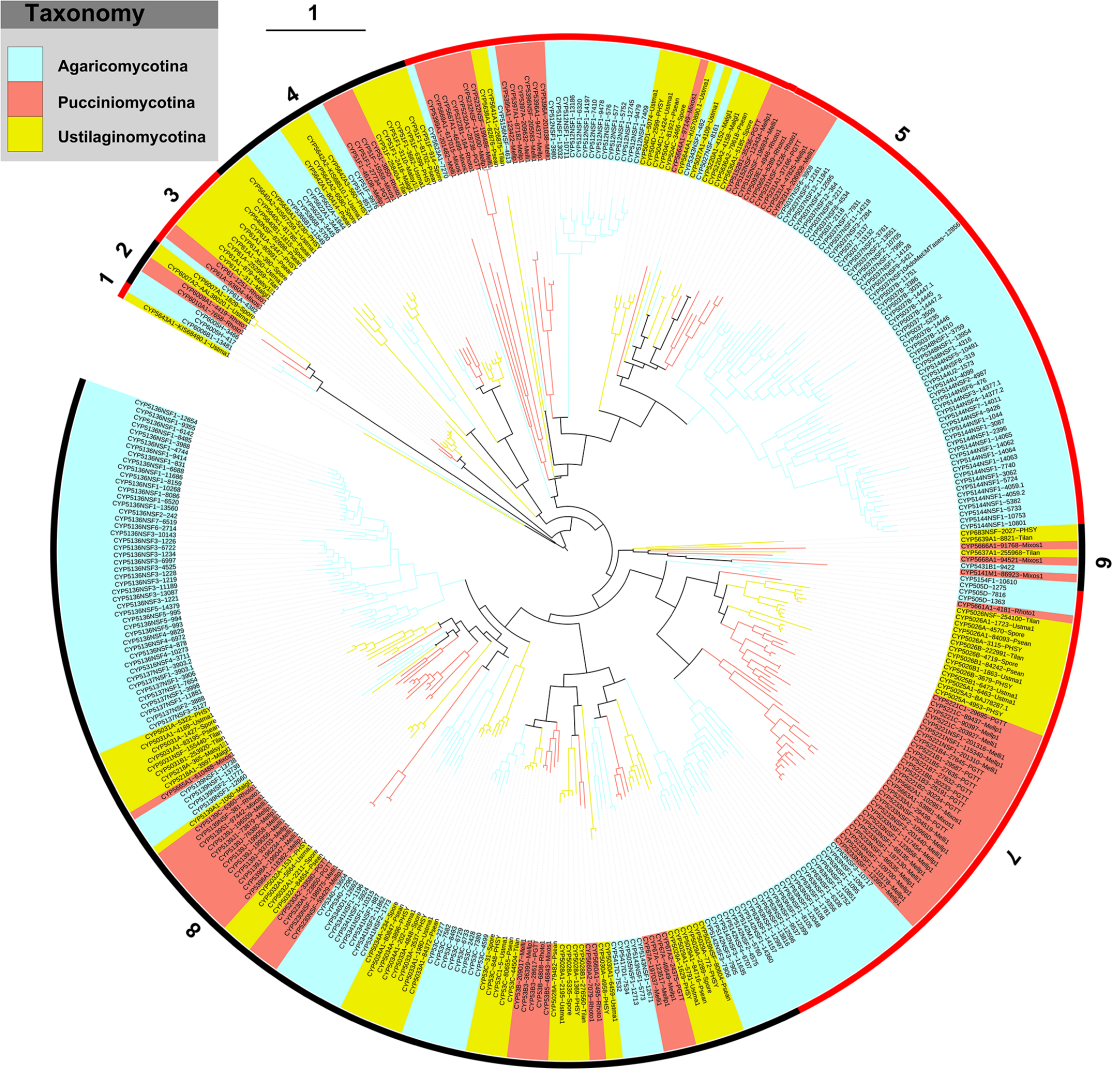
Phylogenetic tree of P450s belonging to basidiomycete biotrophic plant pathogens and non-pathogens used in this study as listed in Table 1. The branches with different colours show their taxonomy, as indicated in the legend. Ancestral branches with children that had identical colours were assigned the same colour as the children. The outer numbers indicate the eight clades, and their ranges are marked by alternating red and black. Each P450 is presented with its family and subfamily following its protein ID (parenthesis). A high-resolution phylogenetic tree is provided as S1 Fig.

The phylogenetic tree of basidiomycete biotrophic plant pathogens was constructed based on their protein sequences (Fig 1). The P450s clustered together on the tree, indicating that they possibly belonged to the same family. The phylogenetic tree played a key role in assigning the family to putative P450s, where the percentage of identity criteria with annotated P450s becomes equal to 40% identity. The alignment helped to find the closest neighbor and thus its percentage identity to its neighbor. Phylogenetic analysis of biotrophic plant pathogen P450s showed numerous branches of P450s in the phylogenetic tree, indicating their highly evolved divergence. This is also reflected in their low percentage identity with homolog P450s belonging to Agaricomycotina species (S2 Table) and the presence of new P450 families and subfamilies (Table 3).

In order to understand the evolution of P450s, a higher level classification of P450s has been proposed [56], indicating their divergence from a common ancestor. In this study, the basidiomycete biotrophic plant pathogens and non-pathogen putative P450s that were annotated in this study were grouped into eight clades based on their phylogenetic relationships (Table 4). Clades 5 and 8 are large branches. Among them, CYP5136, CYP5144, CYP5037 are the very frequently occurring P450s in *A*. *mellea*. The CYP5221 and CYP5231 families were expanded in *P*. *graminis* and *M*. *laricis-populina* (Fig 1). The explosion of these P450 families suggests their specific role in plant pathogens (discussed in the coming sections).

**Table 4:** Clade level classification of P450 families.

Clade	CYP family
1	CYP5643, CYP6006
2	CYP6005, CYP6010, CYP6009, CYP6007
3	CYP61, CYP540
4	CYP5640, CYP5366, CYP5622, CYP5642, CYP51
5	CYP5623, CYP5399, CYP5669, CYP5667, CYP5222, CYP5232, CYP5638, CYP5641, CYP5156, CYP5395, CYP5397, CYP5396, CYP512, CYP504, CYP5664, CYP5644, CYP5343, CYP5027, CYP5220, CYP5636, CYP5152, CYP5093, CYP5065, CYP5231, CYP5037, CYP5348, CYP5144
6	CYP683, CYP5639, CYP5666, CYP5637, CYP5668, CYP5431, CYP5141, CYP5154
7	CYP505, CYP5661, CYP5026, CYP5025, CYP5221, CYP5662, CYP5663, CYP5233, CYP63
8	CYP5142, CYP5029, CYP67, CYP5143, CYP5035, CYP5417, CYP5030, CYP5660, CYP5028, CYP53, CYP5033, CYP5034, CYP5341, CYP5340, CYP5230, CYP5032, CYP5398, CYP5139, CYP5665, CYP5218, CYP5031, CYP5137, CYP5316, CYP5136

### Basidiomycete biotrophic plant pathogens contain unique P450 families

**Fig 2:**
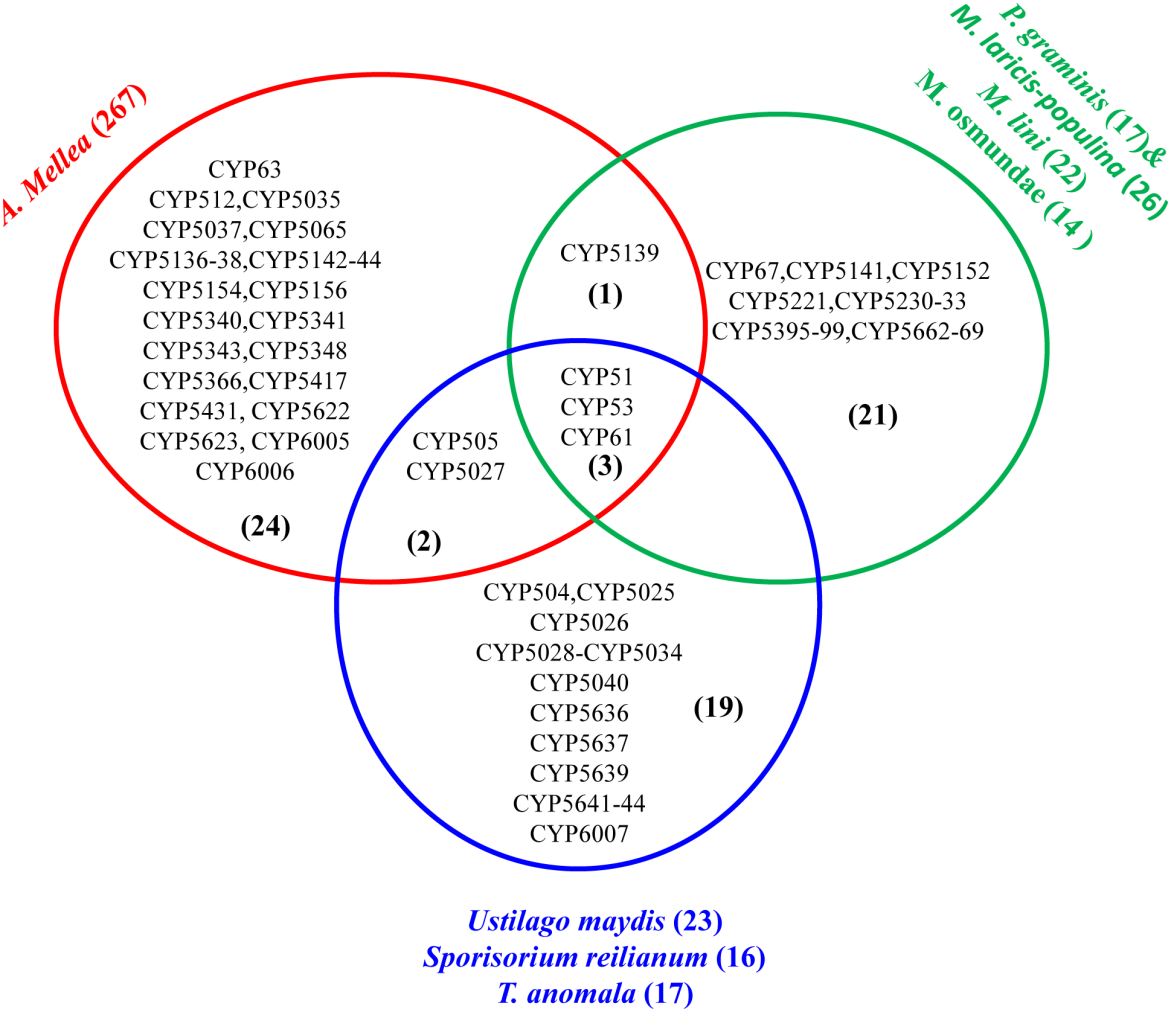
Family level comparative analysis of putative cytochrome P450 monooxygenases between fungal orders represented by *A*. *mellea* (Agaricomycotina), *P*. *graminis*, *M*. *laricis-populina*, *M*. *lini* and *M*. *osmundae* (Pucciniomycotina) and *U*. *maydis*, *S*. *reilianum* and *T*. *anomala* (Ustilaginomycotina). The number in parenthesis indicates P450 family numbers. The number in parenthesis next to each species indicates the total P450 count in the particular species.

**Fig 3:**
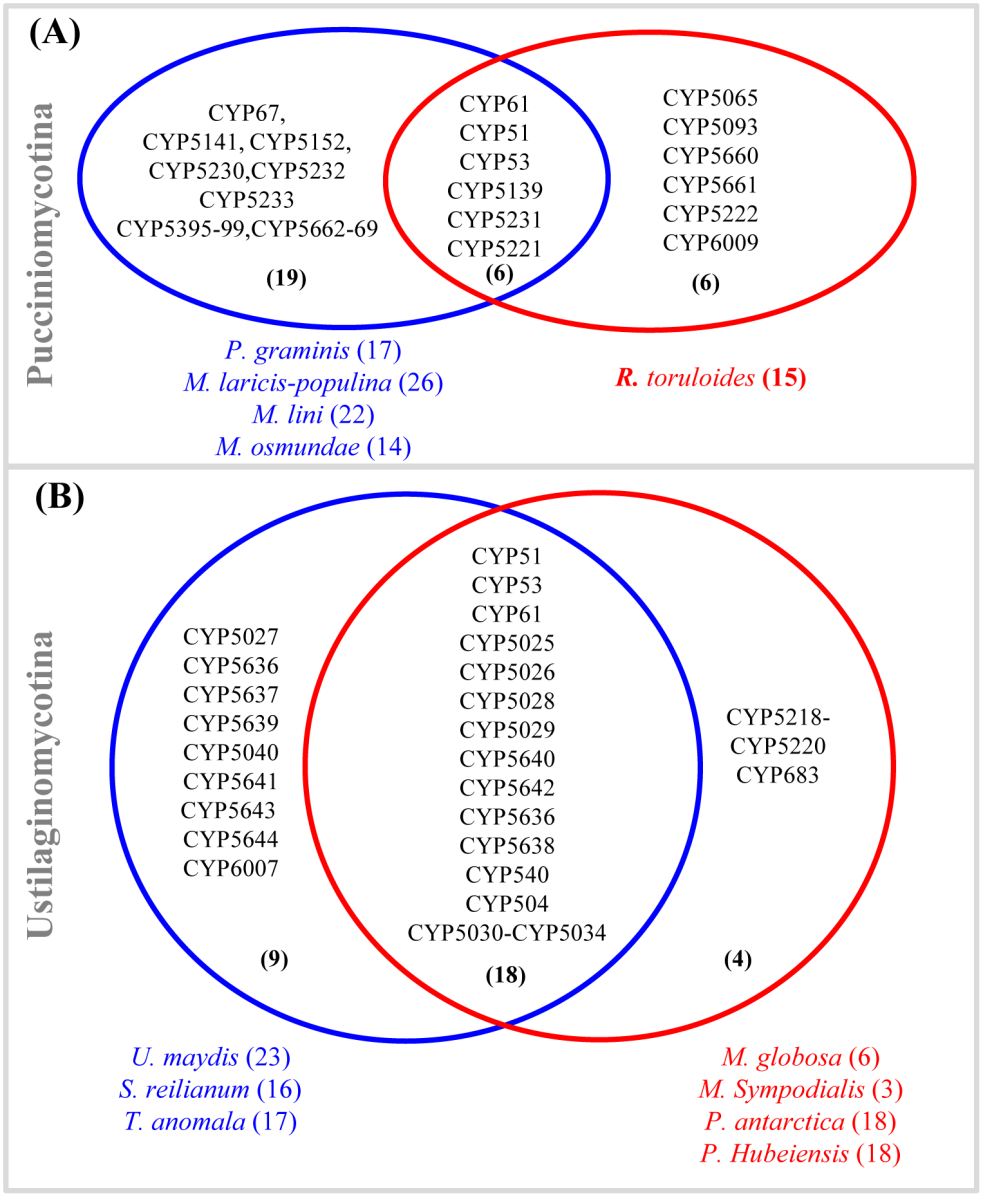
Comparative analysis of member P450s between biotrophic and non-biotrophic basidiomycetes belonging to Pucciniomycotina (A) and Ustilaginomycotina (B). The number in parenthesis indicates P450 family numbers. The number in parenthesis next to each species indicates the total P450 count in the particular species.

Comparison of P450 families between basidiomycete biotrophic plant pathogens revealed the presence of unique P450 families in these species, possibly reflecting the characteristics of their order (Fig 2). As shown in Fig 3, only three P450 families, CYP51, CYP53 and CYP61, are conserved across biotrophic plant pathogens. These P450 families are known to be highly conserved in fungi. *A*. *mellea* shares a single P450 family (CYP5139) with biotrophs belonging to Pucciniomycotina and two P450 families (CYP505 and CYP5027) with biotrophs belong to Ustilaginomycotina. Interestingly, the CYP61 family that is conserved across fungi [16, 17] is missing from *P*. *graminis* and *M*. *laricis-populina*. In a previous study the absence of this P450 family was observed in these organisms [17]. *A*. *mellea* belonging to the Agaricomycotina contains 24 unique P450 families, whereas 21 unique P450 families were found in the Pucciniomycotina species used in this study. Ustilaginomycotina species contain 19 unique P450 families (Fig 2). This clearly suggests that basidiomycete biotrophs belonging to different orders harbour unique P450 families in their genomes.

In order to gain more insight on the unique nature of the biotrophic plant pathogen P450 family contingent, we performed comparative analysis of P450 families between biotrophic plant pathogens and non-biotrophs. Comparison of *A*. *mellea* P450 families with Agaricomycotina saprophytes revealed the presence of five unique P450 families (CYP5417, CYP5431, CYP5622, CYP5623 and CYP6006) in the *A*. *mellea* genome (S3 Table). Interestingly, some of the P450 families were expanded in *A*. *mellea* compared to P450 family members in Agaricomycotina saprophytes. A detailed analysis on *A*. *mellea* P450 families that were expanded is presented in the next section. Comparative analysis revealed the presence of 19 and 9 unique P450 families in biotrophic plant pathogens of Pucciniomycotina and Ustilaginomycotina (Fig 3). The presence of unique P450 families in biotrophs suggests that these P450 families play a role in their adaptation to the biotrophic nature. Future genome sequencing of a greater number of Pucciniomycotina non-pathogens may provide conclusive evidence on unique P450 families in this order of biotrophs, as currently only one species genome is available (Fig 3).

### P450 family expansion in basidiomycete biotrophic plant pathogens

**Fig 4:**
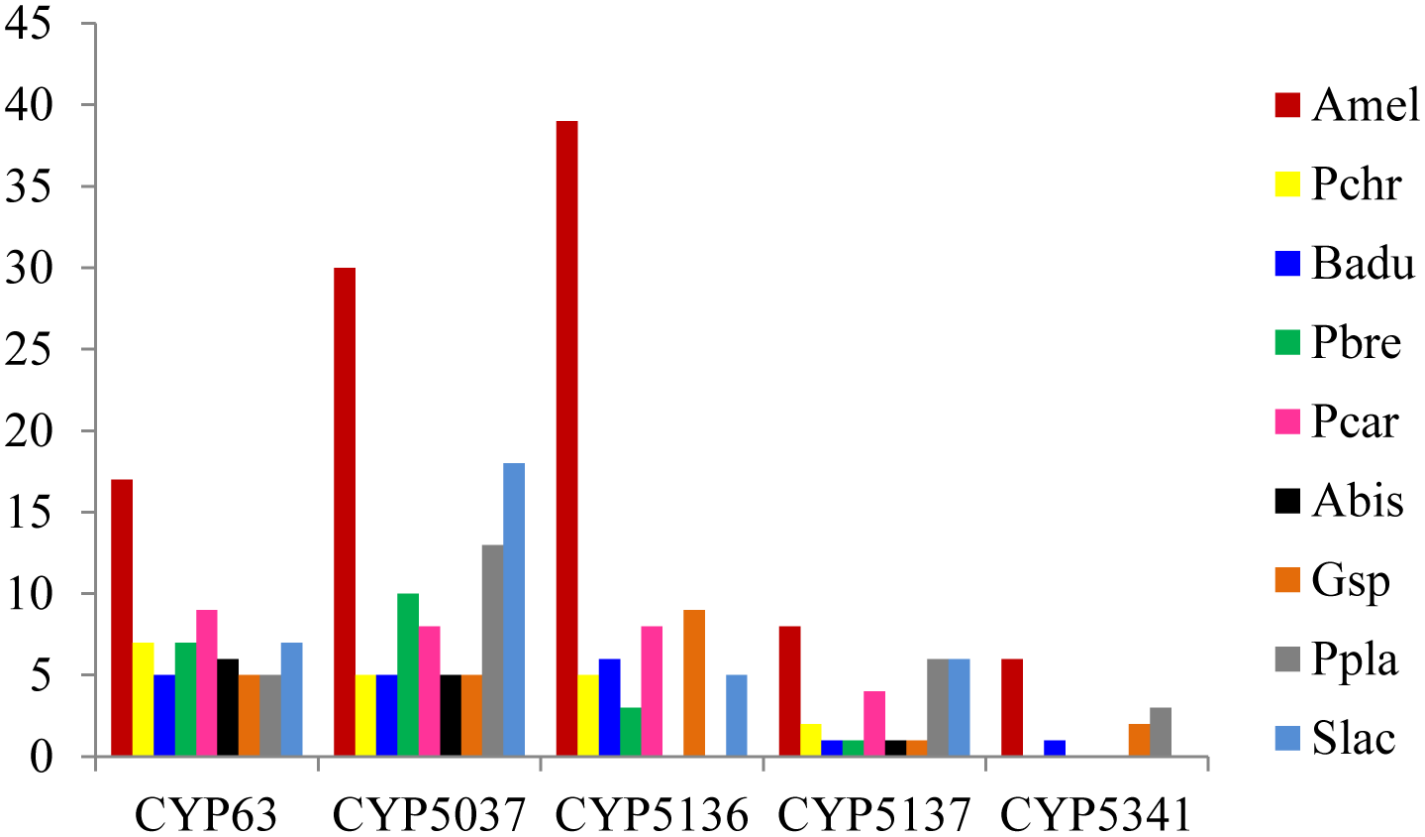
Comparative analysis of putative representatives of enriched P450 families in Agaricomycotina species. The member count in the P450 families that are expanded in *A*. *mellea* is compared with the member count of the same families present in Agaricomycotina saprophytes. The X-axis represents the P450 count and the Y-axis represents P450 families.

Basidiomycetes, especially species belonging to the Agaricomycotina, are well characterized in terms of their P450 annotation and evolutionary analysis [13, 19, 20, 26]. This will give us an advantage when performing a detailed comparison of P450s between saprophytes and biotrophic plant pathogens. Comparative analysis of *A*. *mellea* P450s with P450s of Agaricomycotina saprophytes revealed that the P450 contingent of *A*. *mellea* is unique in terms of P450 family expansion, where certain P450 families were expanded with a very high number of member P450s (Fig 4 and S3 Table).

The P450 families CYP63, CYP5037, CYP5136, CYP5137 and CYP5341 are expanded in *A*. *mellea*. The number of members in these P450 families is as follows: CYP63–17 members; CYP5037–30 members; CYP5136–39 members; CYP5137—eight members and CYP5341- six members. A recent study showed the expansion nature of these P450 families in different Agaricomycotina species [13]. The authors suggested that expansion of these P450 families was due to member P450s’ duplications (paralogous evolution) in order to serve organism adaptation such as colonization of plant material. Comparison of the number of members in these P450 families showed that *A*. *mellea* harbours the largest number of members in these P450 families across Agaricomycotina species used in this study (Fig 4 and S3 Table). Analysis of member P450s suggested that the CYP5221 and CYP5233 P450 families are expanded in *P*. *graminis* and *M*. *laricis-populina* (Fig 1).

### Paralogous evolution of expanded P450 families

It is well known that P450 family expansion is possible because of the duplication of member P450s (paralogous evolution) in an organism [13, 16, 57]. Previous studies on animal (arthropods) [57], fungal [13] and oomycetes [16] P450s revealed the expansion of a large number of P450 families and this expanding nature is attained owing to members’ duplication. In order to understand the mechanism behind the expansion of a large number of P450 families in biotrophic plant pathogens, we proceeded to analyze the paralogous evolution of member P450s, if any.

Paralogous evolution of member P450s can be assessed by analysing the percentage identity among member P450s or the gene structure where duplicated member P450s show conservation in the size of exons *vis à vis* the location of introns. Considering the high percentage identity among the member P450s of expanded P450 families we looked into the gene structure of member P450s and also assessed tandem duplications. Analysis of gene-structure data revealed the conservation of exon size across the members of expanded P450 families (S4 Table). Gene-structure analysis suggested that the CYP63 family in *A*. *mellea* initially contained only two members (orthologs) and one of the members is duplicated 14 times (S4 Table). CYP5136 and CYP5317 family members showed high conservation in exon size and based on the size of the exons it is clear that all the members are paralogs. Four orthologs were observed for the CYP5037 family, where one of the orthologs was duplicated and generated 16 paralogs (S4 Table). Two orthologs were observed for the CYP5341 family, where one ortholog was duplicated twice. The CYP5221 and CYP5233 families contained members that were paralogs, as all of the members showed high conservation in exon size (S4 Table).

From the above data it is clear that members of expanded P450 families of basidiomycete biotrophic plant pathogens were duplicated after speciation (paralogous evolution). Analysis of P450 members of expanded families revealed the presence of a large number of tandemly arranged P450s (S5 Table). Ten members were tandemly arranged in the CYP5136 family (S5 Table). The number of P450s that are tandemly arranged is as follows: CYP5211—seven members; CYP5037—five members; CYP63—four members; CYP5233—three members and CYP5137—two members. Overall, based on conservation in exon size and tandem arrangement of member P450s in the expanded P450 families, we conclude that paralogous evolution of member P450s in these families resulted in expansion of these P450 families.

### Functional role of P450s in basidiomycete biotrophic plant pathogens

The presence of numerous P450s in *A*. *mellea*, expansion of certain P450 families and the presence of distinct P450 families in basidiomycete biotrophic plant pathogens suggest that P450s in these organisms play a key role. Based on homologous P450s characterized in other fungal species, functional analysis for some P450s can be predicted. CYP51, a conserved P450 family across the fungi and also conserved in the species analysed in this study, is involved in the biosynthesis of membrane ergosterol by performing 14α-demethylation of lanosterol [58]. The CYP61 family, which is missing from *M*. *laricis-populina* and *P*. *graminis* (Fig 1), is also involved in membrane ergosterol biosynthesis where it catalyzes C-22 sterol desaturase activity [59]. The absence of CYP61 in *M*. *laricis-populina* and *P*. *graminis* is possibly due to their lifestyle; they are obligate biotrophs that extract essential sterols from plants, as previously suggested [17]. The CYP53 family, also known as benzoate-*p*-hydroxylase, is conserved in biotrophic plant pathogens used in this study (Fig 2). This family is well known for its involvement in detoxification of anti-fungal agents [60]. Study showed that CYP53 family members oxidize benzoate and its derived compounds [61, 62] and plant material stilbene and its derivatives [26]. A recent study suggested that CYP53 family members play a key role in fungal colonization of plant material by detoxification of anti-fungal compounds released by plants or generated during plant material degradation [15]. Also, this study suggested that CYP53 family members play a role in the generation of a secondary metabolite, veratryl alcohol, which is crucial in the degradation of the plant cell wall component, lignin [15]. Based on CYP53 function and the presence of CYP53 members in all four plant pathogens, we conclude that CYP53 members possibly play a key role in colonization of these species on plants through involvement in detoxification of anti-fungal agents and degradation of wood.

Special focus on functional analysis of expanded P450 families in *A*. *mellea* revealed the requirement for expansion of these families in this species. Particularly the CYP63, CYP5037 and CYP5136 families are well known for their catalytic versatility [13]. The CYP512 family, which is expanded in other Agaricomycotina members, also shows catalytic versatility [13]. These families are involved in not only oxidation of xenobiotic compounds, but also in oxidation of key metabolic intermediates in fungi [13]. It is evident that these P450 families possibly play a key role in *A*. *mellea* towards successful colonization on plants (infection), hence these P450 families are expanded in this species. A putative ortholog of the CYP504 family is present only in *U*. *maydis* involved in oxidation of phenylacetate and its derived compounds [63, 64]. Phenylacetate is a plant growth hormone [65] and oxidation of this and its derivatives by CYP504 clearly suggests that after infection this P450 may be involved in interfering with the growth of plants by oxidizing the plant growth hormone by *U*. *maydis*. It is noteworthy that the CYP504 family usually presents in most of the plant pathogens [17]. CYP505 family members are involved in oxidation of fatty acids [66] and their role in the generation of mycotoxin fumonisin has also been elucidated [67]. The CYP5138, CYP5139 and CYP5144 families were shown to oxidize xenobiotic compounds [13, 37]. The fused P450s belonging to CYP6000 series contain the N-terminal heme-peroxidase motif and C-terminal heme-domain characteristic of P450s [16]. As CYP6005-CYP6007 family members also contain the same motifs as CYP6001 [68], it is possible that P450s belong to CYP6005-CYP6007 families are involved in oxidation of fatty acids. Overall, based on the above available homologous P450s functions, we conclude that P450s in these plant pathogens possibly play a key role not only in their primary metabolism, but also in successful colonization on living plants by degradation of plant material, detoxification of plant defence chemicals and oxidation of xenobiotic compounds. Functional characterization of P450s in these organisms will provide more insight into their role.

## Conclusions

It is well known that ecological niches including the host (a parasite or a symbiont or a commensal) play a key role in shaping the genome content of an organism. Fungi, especially saprophytic species belonging to Agaricomycotina, play a key role in the carbon cycle by degradation of one of the most abundant photosynthetically fixed carbon sources, i.e. plant material. Because of their adaptation to the same ecological niche, similar P450 family types were observed, despite a few differences in P450 families among Agaricomycotina saprophytes. In this study, we present a good example of the influence of ecological niches on the P450 patterns of an organism. Analysis of putative P450s in basidiomycete biotrophic plant pathogens revealed the presence of unique P450 families, possibly reflecting the characteristics of their order. The presence of unique P450 families in these biotrophic plant pathogens serves as good evidence of how a host can influence shaping the P450 content of an organism. These unique P450 family members might play a key role in successful infection of the host. It is noteworthy that P450 patterns in basidiomycete plant pathogens are poorly studied compared to their counterpart ascomycete plant pathogens. This study is the first report on comparative analysis of P450s in basidiomycete biotrophic plant pathogens at order level.

## Supporting Information

S1 Fig A high-resolution phylogenetic tree. (PDF)

S1 Table Cytochrome P450 monooxygenases in basidiomycete biotrophic plant pathogens and non-pathogens annotated in this study. Each species P450s was presented with its protein IDs that were identified in our analysis at species individual databases listed in Table 1. The number in parenthesis next to the species name is the total P450 count in the species.
(DOCX)

S2 Table Annotation of cytochrome P450 monooxygenases in basidiomycete biotrophic plant pathogens and non-pathogens used in this study. P450 sequences for *U*. *maydis*, *M*. *globosa* and *P*. *graminis* were retrieved from the Cytochrome P450 Homepage [42] and corresponding protein IDs were assigned as per their databases at the Joint Genome Institute (Table 1). Protein IDs for reference P450s (homolog P450s with highest percent identity) from the Cytochrome P450 Homepage [42] are not shown in the table, considering their availability on the webpages listed in Table 1.
(DOCX)

S3 Table Family-level comparative analysis of cytochrome P450 monooxygenases in Agaricomycotina species. (DOCX)

S4 Table Gene-structure analysis of member P450s belonging to the expanded P450 families in *A*. *mellea* (CYP63, CYP5136, CYP5137, CYP5037 and CYP5341), *M*. *laricis-populina* (CYP5223) and *P*. *graminis* (CYP5221). Gene-structure analysis was carried out by analysis of conservation of exon size across the member P450s. The size of each exon in member P450s is arranged in a way that reflects the conservation pattern. Possible orthologs in each family are also shown in the table.
(XLSX)

S5 Table Analysis of tandem gene duplications in expanded P450 families. The member P450s’ genomic localization such as scaffold/node and the DNA region (start and end) are shown in the table. P450s are presented with their protein IDs. Member P450s that are tandemly duplicated are highlighted in red font.
(DOCX)
