# Supplementary figures and images for: Genome-Wide Annotation and Comparative Analysis of Cytochrome P450 Monooxygenases in Basidiomycete Biotrophic Plant Pathogens
Source: PLoS One. 2015 Nov 4;10(11):e0142100. doi: 10.1371/journal.pone.0142100 (PMC4633277; doi:10.1371/journal.pone.0142100)

## 1

## Ustilaginomycotina

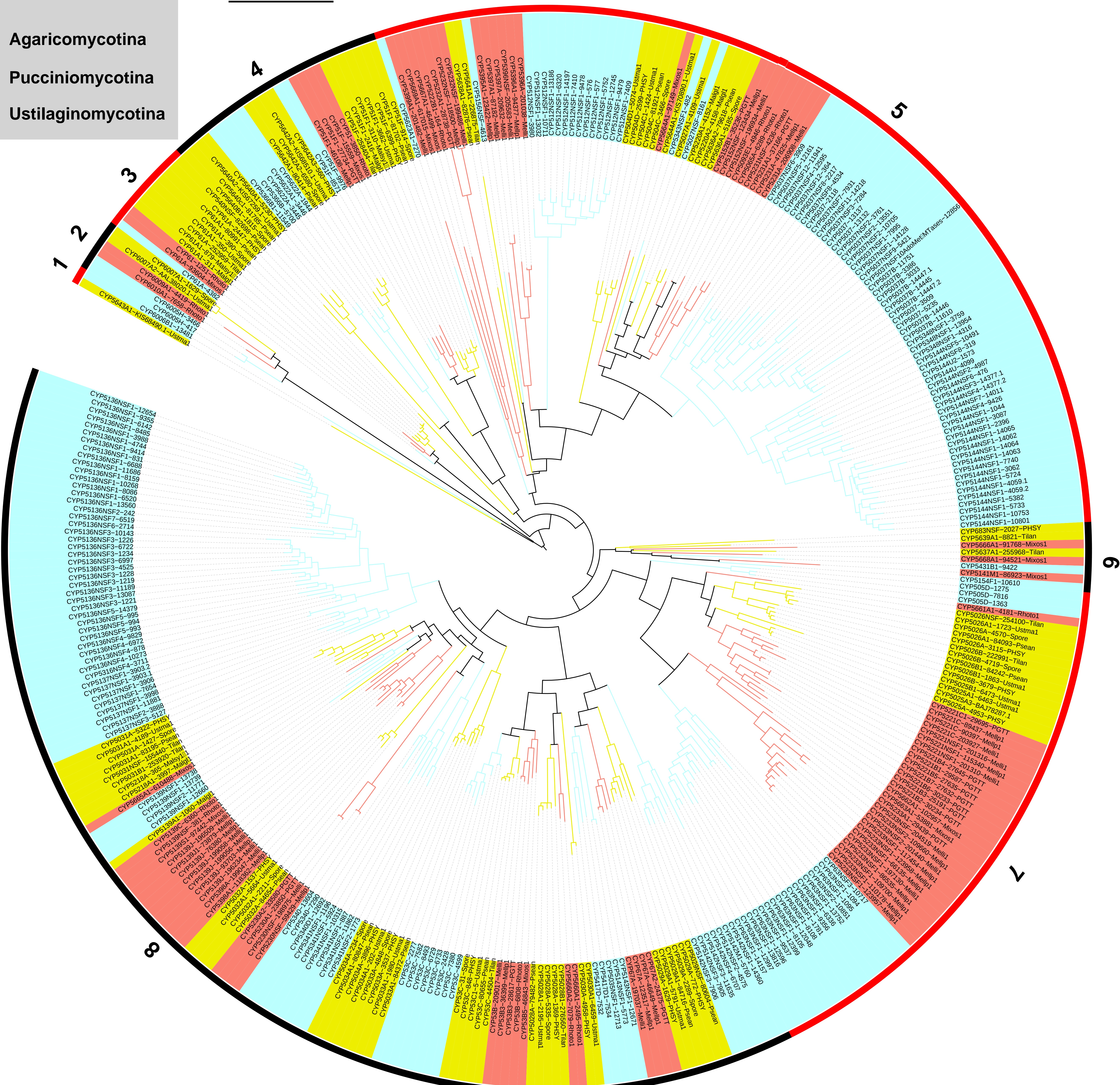

Supplement: S1 Fig — (PDF) [file pone.0142100.s001.pdf]
