# Supplementary material for: Genome-Wide Annotation and Comparative Analysis of Cytochrome P450 Monooxygenases in Basidiomycete Biotrophic Plant Pathogens
Source: PLoS One. 2015 Nov 4;10(11):e0142100. doi: 10.1371/journal.pone.0142100 (PMC4633277; doi:10.1371/journal.pone.0142100)
Supplement: S1 Table — Each species P450s was presented with its protein IDs that were identified in our analysis at species individual databases listed in Table 1. The number in parenthesis next to the species name is the total P450 count in the species. (DOCX) [file pone.0142100.s002.docx]

| ***Armillaria mellea* P450s (267)** | | | | | | | |
| --- | --- | --- | --- | --- | --- | --- | --- |
| 8161 | 1219 | 14011 | 10315 | 9721 | 8485 | 14010 | 7607 |
| 11751 | 1221 | 4613 | 5924 | 3289 | 9273 | 1595 | 7608 |
| 14445 | 1226 | 9976 | 1196 | 9720 | 993 | 4144 | 7881 |
| 14446 | 1228 | 8571 | 11862 | 11344 | 14302 | 743 | 7906 |
| 3033 | 13087 | 2277 | 1773 | 10748 | 2382 | 7380 | 7951 |
| 14128 | 6997 | 2428 | 12692 | 2053 | 2713 | 7935 | 8685 |
| 7995 | 6972 | 4599 | 12671 | 8641 | 273 | 13132 | 8797 |
| 12856 | 878 | 6729 | 5773 | 5405 | 8686 | 364 | 9414 |
| 14218 | 994 | 6733 | 12713 | 1044 | 10814 | 5152 | 9426 |
| 11941 | 995 | 7582 | 7532 | 11610 | 7749 | 1 | 9479 |
| 13551 | 2714 | 8493 | 7534 | 14447 | 1232 | 9829 | 9525 |
| 3761 | 6519 | 3466 | 5760 | 2118 | 7290 | 6336 |  |
| 7284 | 11881 | 417 | 9422 | 3509 | 13324 | 9537 |  |
| 12595 | 3903.1 | 4382 | 1573 | 5235 | 12929 | 5700 |  |
| 12161 | 3903.2 | 1094 | 12596 | 3386 | 6735 | 1095 |  |
| 3909 | 3906 | 12048 | 13481 | 10705 | 261 | 9108 |  |
| 7931 | 3998 | 12399 |  | 13137 | 11492 | 10068 |  |
| 2217 | 7654 | 12997 |  | 480 | 6700 | 10268 |  |
| 4534 | 3888 | 13752 |  | 2728 | 14306 | 10551 |  |
| 5421 | 5127 | 13816 |  | 5093 | 171 | 10611 |  |
| 1275 | 12660 | 14157 |  | 12893 | 10801 | 10753 |  |
| 1363 | 13739 | 1781 |  | 3980 | 14062 | 11148 |  |
| 7816 | 11771 | 8105 |  | 7409 | 14064 | 12563 |  |
| 10711 | 14360 | 8108 |  | 9478 | 14066 | 9616 |  |
| 12745 | 4575 | 9356 |  | 577 | 3062 | 13604 |  |
| 13032 | 11635 | 10851 |  | 576 | 5382 | 14063 |  |
| 13186 | 7905 | 10717 |  | 7533 | 8391 | 1721 |  |
| 14197 | 14065 | 5298 |  | 10143 | 9608 | 2267 |  |
| 5752 | 2396 | 13954 |  | 10273 | 319 | 2295 |  |
| 7410 | 3087 | 3759 |  | 11189 | 12429 | 3465 |  |
| 11686 | 4059.1 | 4316 |  | 1234 | 8523 | 4099 |  |
| 13560 | 4059.2 | 1944 |  | 12646 | 2727 | 4383 |  |
| 3988 | 5724 | 3445 |  | 12654 | 352 | 479 |  |
| 4744 | 5733 | 3446 |  | 14379 | 1236 | 481 |  |
| 6520 | 7740 | 11549 |  | 2417 | 13738 | 5123 |  |
| 8086 | 4987 | 482 |  | 3711 | 3410 | 5397 |  |
| 8159 | 14377.1 | 4550 |  | 4524 | 9535 | 629 |  |
| 831 | 14377.2 | 10610 |  | 4525 | 165 | 6320 |  |
| 9355 | 10491 | 7270 |  | 6142 | 1003 | 6707 |  |
| 242 | 476 | 887 |  | 6688 | 11698 | 6722 |  |
|  |  |  |  |  |  |  |  |
|  | |  |  |  |  |  |  |
|  | |  |  |  |  |  |  |
|  | |  |  |  |  |  |  |
|  | |  |  |  |  |  |  |
|  | |  |  |  |  |  |  |
| ***Malassezia globosa* (6)** | | ***Malassezia sympodialis* ATCC 42132 (3)** | ***Pseudozyma antarctica* T-34 (18)** | ***Pseudozyma hubeiensis* SY62 (18)** | ***Sporisorium reilianum* SRZ2 (16)** | ***Tilletiaria anomala* UBC 951 v1.0 (17)** | ***Ustilago maydis***  **P450s (23)** |
| 1060 | | 3110 | 79482 | 772 | 31 | 276560 | 6463 |
| 3997 | | 879 | 80655 | 1537 | 3466 | 44504 | 6473 |
| 2416 | | 365 | 80604 | 2027 | 5335 | 255968 | 1723 |
| 4152 | |  | 81785 | 3537 | 6580 | 155440 | 1863 |
| 4158 | |  | 82698 | 4958 | 914 | 253920 | 4109 |
| 311 | |  | 81921 | 848 | 1815 | 258404 | 2195 |
|  | |  | 80847 | 1369 | 1427 | 226875 | 5791 |
|  | |  | 81702 | 3896 | 2211 | 8821 | 6459 |
|  | |  | 84372 | 4953 | 2289 | 222991 | 4189 |
|  | |  | 83195 | 5322 | 4848 | 254100 | 5664 |
|  | |  | 84654 | 6399 | 5185 | 252959 | 1980 |
|  | |  | 84716 | 1629 | 4570 | 230266 | 202 |
|  | |  | 80414 | 2447 | 4719 | 276164 | 1424 |
|  | |  | 84093 | 2599 | 390 | 64145 | 5074 |
|  | |  | 84242 | 3115 | 234 | 230244 | 3662 |
|  | |  | 79618 | 3679 | 1629 | 259198 | 5 |
|  | |  | 80991 | 560 |  | 263488 | 350 |
|  | |  | 82876 | 5230 |  |  | BAJ78287.1 |
|  | |  |  |  |  |  | KIS67259.1 |
|  | |  |  |  |  |  | KIS68510.1 |
|  | |  |  |  |  |  | KIS68490.1 |
|  | |  |  |  |  |  | KIS70890.1 |
|  | |  |  |  |  |  | AAL38020.1 |
|  | |  |  |  |  |  |  |
|  | |  |  |  |  |  |  |
| ***Melampsora laricis-populina* P450s (28)** | | ***Melampsora lini*** **CH5** **(22)** | ***Puccinia graminis***  **P450s (17)** | ***Mixia osumundae* (14)** | ***Rhodosporidium toruloides* NP11** **(15)** |  |  |
| 123434 | | 209017 | 35236 | 54045 | 2495 |  |  |
| 46108 | | 201316 | 27734 | 86923 | 6808 |  |  |
| 89437 | | 197130 | 29587 | 102957 | 4236 |  |  |
| 90397 | | 199058 | 30234 | 46843 | 4946 |  |  |
| 115340 | | 199047 | 25191 | 53881 | 3850 |  |  |
| 59429 | | 209632 | 27645 | 97149 | 381 |  |  |
| 47825 | | 201482 | 27635 | 610488 | 4126 |  |  |
| 118500 | | 204519 | 30233 | 15559 | 4181 |  |  |
| 109700 | | 196934 | 27632 | 91768 | 6289 |  |  |
| 110178 | | 205583 | 29695 | 46499 | 6360 |  |  |
| 117454 | | 206908 | 23850 | 94521 | 7079 |  |  |
| 123957 | | 198975 | 33580 | 93604 | 1251 |  |  |
| 123958 | | 201310 | 37166 | 97442 | 164 |  |  |
| 66135 | | 201440 | 28738 | 617015 | 4419 |  |  |
| 86535 | | 197037 | 29439 |  | 7658 |  |  |
| 109660 | | 199056 | 28617 |  |  |  |  |
| 36399 | | 196234 | 24375 |  |  |  |  |
| 123517 | | 201038 |  |  |  |  |  |
| 46649 | | 196509 |  |  |  |  |  |
| 73879 | | 199486 |  |  |  |  |  |
| 76380 | | 203927 |  |  |  |  |  |
| 93703 | | 202499 |  |  |  |  |  |
| 118362 | |  |  |  |  |  |  |
| 94377 | |  |  |  |  |  |  |
| 123422 | |  |  |  |  |  |  |
| 87182 | |  |  |  |  |  |  |
| 92310 | |  |  |  |  |  |  |
| 75472 | |  |  |  |  |  |  |
|  | |  |  |  |  |  |  |
