# Supplementary material for: Genome-Wide Annotation and Comparative Analysis of Cytochrome P450 Monooxygenases in Basidiomycete Biotrophic Plant Pathogens
Source: PLoS One. 2015 Nov 4;10(11):e0142100. doi: 10.1371/journal.pone.0142100 (PMC4633277; doi:10.1371/journal.pone.0142100)
Supplement: S2 Table — P450 sequences for U. maydis, M. globosa and P. graminis were retrieved from the Cytochrome P450 Homepage [42] and corresponding protein IDs were assigned as per their databases at the Joint Genome Institute (Table 1). Protein IDs for reference P450s (homolog P450s with highest percent identity) from the Cytochrome P450 Homepage [42] are not shown in the table, considering their availability on the webpages listed in Table 1. (DOCX) [file pone.0142100.s003.docx]

| ***Armillaria mellea*** | | | | | | | |
| --- | --- | --- | --- | --- | --- | --- | --- |
|  |  |  |  | | **Reference P450s** | | |
| **P450 name** | **Protein ID** | **Species** | **Identity %** | | **P450 name** | **Protein ID** | **Species** |
| CYP5136NSF1 | 831 | *A. mellea* | 83.64 | | CYP5136NSF1 | 4744 | *A. mellea* |
| CYP5136NSF1 | 4744 | *A. mellea* | 41 | | CYP5136A1 |  | *P. chrysosporium* |
| CYP5136NSF1 | 3988 | *A. mellea* | 65.53 | | CYP5136NSF1 | 831 | *A. mellea* |
| CYP5136NSF1 | 9355 | *A. mellea* | 65.06 | | CYP5136NSF1 | 831 | *A. mellea* |
| CYP5136NSF1 | 6520 | *A. mellea* | 66.25 | | CYP5136NSF1 | 831 | *A. mellea* |
| CYP5136NSF1 | 8086 | *A. mellea* | 72.51 | | CYP5136NSF1 | 831 | *A. mellea* |
| CYP5136NSF1 | 8159 | *A. mellea* | 71.09 | | CYP5136NSF1 | 831 | *A. mellea* |
| CYP5136NSF1 | 11686 | *A. mellea* | 64.97 | | CYP5136NSF1 | 831 | *A. mellea* |
| CYP5136NSF1 | 13560 | *A. mellea* | 57.03 | | CYP5136NSF1 | 8159 | *A. mellea* |
| CYP5136NSF6 | 2714 | *A. mellea* | 44.21 | | CYP5136NSF1 | 831 | *A. mellea* |
| CYP5136NSF7 | 6519 | *A. mellea* | 54.65 | | CYP5136NSF1 | 831 | *A. mellea* |
| CYP5136NSF2 | 242 | *A. mellea* | 55.19 | | CYP5136NSF7 | 6519 | *A. mellea* |
| CYP5136NSF3 | 1221 | *A. mellea* | 44.56 | | CYP5136NSF1 | 831 | *A. mellea* |
| CYP5136NSF3 | 13087 | *A. mellea* | 73.1 | | CYP5136NSF3 | 1221 | *A. mellea* |
| CYP5136NSF3 | 6997 | *A. mellea* | 57.7 | | CYP5136NSF3 | 1221 | *A. mellea* |
| CYP5136NSF3 | 1226 | *A. mellea* | 71.61 | | CYP5136NSF3 | 6997 | *A. mellea* |
| CYP5136NSF3 | 1228 | *A. mellea* | 70.79 | | CYP5136NSF3 | 6997 | *A. mellea* |
| CYP5136NSF3 | 1219 | *A. mellea* | 57.81 | | CYP5136NSF3 | 6997 | *A. mellea* |
| CYP5136NSF4 | 6972 | *A. mellea* | 44 | | CYP5136A2 |  | *P. chrysosporium* |
| CYP5136NSF4 | 878 | *A. mellea* | 86.98 | | CYP5136NSF4 | 6972 | *A. mellea* |
| CYP5136NSF5 | 995 | *A. mellea* | 40.83 | | CYP5136NSF1 | 13560 | *A. mellea* |
| CYP5136NSF5 | 994 | *A. mellea* | 56.55 | | CYP5136NSF5 | 995 | *A. mellea* |
| CYP5136NSF3 | 10143 | *A. mellea* | 73.37 | | CYP5136NSF3 | 1226 | *A. mellea* |
| CYP5136NSF3 | 6722 | *A. mellea* | 76.09 | | CYP5136NSF3 | 10143 | *A. mellea* |
| CYP5136NSF3 | 1234 | *A. mellea* | 71.74 | | CYP5136NSF3 | 10143 | *A. mellea* |
| CYP5136NSF3 | 11189 | *A. mellea* | 66.67 | | CYP5136NSF3 | 13087 | *A. mellea* |
| CYP5136NSF3 | 4525 | *A. mellea* | 65.6 | | CYP5136NSF3 | 13087 | *A. mellea* |
| CYP5136NSF4 | 3711 | *A. mellea* | 62.5 | | CYP5136NSF4 | 6972 | *A. mellea* |
| CYP5136NSF4 | 10273 | *A. mellea* | 69.5 | | CYP5136NSF4 | 6972 | *A. mellea* |
| CYP5136NSF4 | 9829 | *A. mellea* | 73.9 | | CYP5136NSF4 | 6972 | *A. mellea* |
| CYP5136NSF1 | 6142 | *A. mellea* | 70.17 | | CYP5136NSF1 | 9355 | *A. mellea* |
| CYP5136NSF1 | 12654 | *A. mellea* | 74.0 | | CYP5136NSF1 | 9355 | *A. mellea* |
| CYP5136NSF1 | 8485 | *A. mellea* | 78.04 | | CYP5136NSF1 | 4744 | *A. mellea* |
| CYP5136NSF1 | 9414 | *A. mellea* | 84.66 | | CYP5136NSF1 | 831 | *A. mellea* |
| CYP5136NSF1 | 10268 | *A. mellea* | 67.12 | | CYP5136NSF1 | 6520 | *A. mellea* |
| CYP5136NSF1 | 6688 | *A. mellea* | 90.24 | | CYP5136NSF1 | 8159 | *A. mellea* |
| CYP5136NSF5 | 14379 | *A. mellea* | 63.32 | | CYP5136NSF5 | 995 | *A. mellea* |
| CYP5136NSF5 | 993 | *A. mellea* | 64.98 | | CYP5136NSF5 | 995 | *A. mellea* |
| CYP5137NSF1 | 11881 | *A. mellea* | 58.35 | | CYP5137NSF1 | 3998 | *A. mellea* |
| CYP5137NSF1 | 3903.1 | *A. mellea* | 77.11 | | CYP5137NSF1 | 3906 | *A. mellea* |
| CYP5137NSF1 | 3903.2 | *A. mellea* | 72.47 | | CYP5137NSF1 | 3903.1 | *A. mellea* |
| CYP5137NSF1 | 3906 | *A. mellea* | 57.44 | | CYP5137NSF1 | 3998 | *A. mellea* |
| CYP5137NSF1 | 3998 | *A. mellea* | 43 | | CYP5137A1 |  | *P. chrysosporium* |
| CYP5137NSF3 | 5127 | *A. mellea* | 45.77 | | CYP5137NSF1 | 3998 | *A. mellea* |
| CYP5137NSF1 | 7654 | *A. mellea* | 58.58 | | CYP5137NSF1 | 3998 | *A. mellea* |
| CYP5137NSF2 | 3888 | *A. mellea* | 50 | | CYP5137A2 |  | *P. chrysosporium* |
| CYP5341NSF1 | 1196 | *A. mellea* | 48.38 | | CYP5341B1v2 |  | *P. placenta* |
| CYP5341NSF1 | 887 | *A. mellea* | 46.24 | | CYP5341NSF1 | 1196 | *A. mellea* |
| CYP5341NSF1 | 5924 | *A. mellea* | 40.44 | | CYP5341NSF1 | 887 | *A. mellea* |
| CYP5341NSF1 | 10315 | *A. mellea* | 52.12 | | CYP5341NSF1 | 887 | *A. mellea* |
| CYP5341NSF2 | 1773 | *A. mellea* | 54.64 | | CYP5341A2 |  | *P. placenta* |
| CYP5341NSF2 | 11862 | *A. mellea* | 73.49 | | CYP5341NSF2 | 1773 | *A. mellea* |
| CYP5139NSF1 | 13739 | *A. mellea* | 48 | | CYP5139A1 |  | *P. chrysosporium* |
| CYP5139NSF2 | 11771 | *A. mellea* | 43 | | CYP5139A1 |  | *P. chrysosporium* |
| CYP5139NSF1 | 13738 | *A. mellea* | 58.44 | | CYP5139NSF1 | 13739 | *A. mellea* |
| CYP5138NSF1 | 12660 | *A. mellea* | 43 | | CYP5138A1 |  | *P. chrysosporium* |
| CYP5143NSF1 | 12671 | *A. mellea* | 43.2 | | CYP5143B1 |  | *B. adusta* |
| CYP5143NSF1 | 5773 | *A. mellea* | 88.97 | | CYP5143NSF1 | 12671 | *A. mellea* |
| CYP63NSF1 | 1094 | *A. mellea* | 47.68 | | CYP63NSF3 | 10717 | *A. mellea* |
| CYP63NSF1 | 12596 | *A. mellea* | 65.51 | | CYP63NSF1 | 8108 | *A. mellea* |
| CYP63NSF1 | 12048 | *A. mellea* | 68.82 | | CYP63NSF1 | 1781 | *A. mellea* |
| CYP63NSF1 | 12399 | *A. mellea* | 78.67 | | CYP63NSF1 | 8105 | *A. mellea* |
| CYP63NSF1 | 12997 | *A. mellea* | 67.8 | | CYP63NSF1 | 1781 | *A. mellea* |
| CYP63NSF1 | 13752 | *A. mellea* | 56.56 | | CYP63NSF1 | 1781 | *A. mellea* |
| CYP63NSF1 | 13816 | *A. mellea* | 68.98 | | CYP63NSF1 | 1781 | *A. mellea* |
| CYP63NSF1 | 14157 | *A. mellea* | 65.67 | | CYP63NSF1 | 1781 | *A. mellea* |
| CYP63NSF1 | 1781 | *A. mellea* | 71.84 | | CYP63NSF1 | 8105 | *A. mellea* |
| CYP63NSF1 | 8105 | *A. mellea* | 52 | | CYP63A4 |  | *P. chrysosporium* |
| CYP63NSF1 | 8108 | *A. mellea* | 75.91 | | CYP63NSF1 | 1781 | *A. mellea* |
| CYP63NSF1 | 9356 | *A. mellea* | 64.56 | | CYP63NSF1 | 1781 | *A. mellea* |
| CYP63NSF2 | 10851 | *A. mellea* | 48.83 | | CYP63NSF1 | 8108 | *A. mellea* |
| CYP63NSF3 | 10717 | *A. mellea* | 48.52 | | CYP63NSF1 | 1781 | *A. mellea* |
| CYP63NSF1 | 9537 | *A. mellea* | 73.09 | | CYP63NSF1 | 12399 | *A. mellea* |
| CYP63NSF1 | 6336 | *A. mellea* | 95.24 | | CYP63NSF1 | 13752 | *A. mellea* |
| CYP63NSF1 | 1095 | *A. mellea* | 50.97 | | CYP63NSF1 | 1094 | *A. mellea* |
| CYP505D | 7816 | *A. mellea* | 57 | | CYP505D6 |  | *P. chrysosporium* |
| CYP505D | 1363 | *A. mellea* | 85.88 | | CYP505D | 7816 | *A. mellea* |
| CYP505D | 1275 | *A. mellea* | 55.45 | | CYP505D | 7816 | *A. mellea* |
| CYP53C | 4599 | *A. mellea* | 64 | | CYP53C2 |  | *P. chrysosporium* |
| CYP53C | 6729 | *A. mellea* | 57 | | CYP53C2 |  | *P. chrysosporium* |
| CYP53C | 2428 | *A. mellea* | 60 | | CYP53C2 |  | *P. chrysosporium* |
| CYP53C | 6733 | *A. mellea* | 59.21 | | CYP53C | 4599 | *A. mellea* |
| CYP53C | 7582 | *A. mellea* | 68.67 | | CYP53C | 6733 | *A. mellea* |
| CYP53C | 2277 | *A. mellea* | 84.46 | | CYP53C | 7582 | *A. mellea* |
| CYP53C | 8493 | *A. mellea* | 74.19 | | CYP53C | 7582 | *A. mellea* |
| CYP53C | 97.65 | *A. mellea* | 97.65 | | CYP53C | 4599 | *A. mellea* |
| CYP5142NSF1 | 14360 | *A. mellea* | 43 | | CYP5142E1 |  | *P. chrysosporium* |
| CYP5142NSF2 | 4575 | *A. mellea* | 43 | | CYP5142E1 |  | *P. chrysosporium* |
| CYP5142NSF3 | 7905 | *A. mellea* | 41 | | CYP5142E1 |  | *P. chrysosporium* |
| CYP5142NSF3 | 11635 | *A. mellea* | 73.72 | | CYP5142NSF3 | 7905 | *A. mellea* |
| CYP5142NSF3 | 7906 | *A. mellea* | 67.85 | | CYP5142NSF3 | 11635 | *A. mellea* |
| CYP5144NSF4 | 6707 | *A. mellea* | 47.73 | | CYP5142NSF3 | 7905 | *A. mellea* |
| CYP5144NSF1 | 2396 | *A. mellea* | 43 | | CYP5144C1 |  | *P. chrysosporium* |
| CYP5144NSF1 | 5733 | *A. mellea* | 70.55 | | CYP5144NSF1 | 2396 | *A. mellea* |
| CYP5144NSF1 | 14065 | *A. mellea* | 71.67 | | CYP5144NSF1 | 2396 | *A. mellea* |
| CYP5144NSF1 | 4059.1 | *A. mellea* | 69.51 | | CYP5144NSF1 | 5733 | *A. mellea* |
| CYP5144NSF1 | 4059.2 | *A. mellea* | 80.49 | | CYP5144NSF1 | 4059.1 | *A. mellea* |
| CYP5144NSF2 | 4987 | *A. mellea* | 42.35 | | CYP5144NSF1 | 4059.2 | *A. mellea* |
| CYP5144NSF1 | 5724 | *A. mellea* | 75.63 | | CYP5144NSF1 | 4059.2 | *A. mellea* |
| CYP5144NSF1 | 7740 | *A. mellea* | 74.2 | | CYP5144NSF1 | 2396 | *A. mellea* |
| CYP5144NSF1 | 3087 | *A. mellea* | 69.81 | | CYP5144NSF1 | 5733 | *A. mellea* |
| CYP5144NSF3 | 14377.1 | *A. mellea* | 44.86 | | CYP5144NSF2 | 4987 | *A. mellea* |
| CYP5144NSF4 | 14377.2 | *A. mellea* | 52.96 | | CYP5144NSF3 | 14377.1 | *A. mellea* |
| CYP5144NSF5 | 10491 | *A. mellea* | 46.11 | | CYP5144NSF2 | 4987 | *A. mellea* |
| CYP5144NSF6 | 476 | *A. mellea* | 45.73 | | CYP5144NSF1 | 4059.1 | *A. mellea* |
| CYP5144NSF7 | 14011 | *A. mellea* | 52.65 | | CYP5144NSF3 | 14377.1 | *A. mellea* |
| CYP5144U2 | 1573 | *A. mellea* |  | | Named by Prof David R Nelson | | |
| CYP5144U | 4099 | *A. mellea* | 68.7 | | CYP5144U2 | 1573 | *A. mellea* |
| CYP5144NSF8 | 319 | *A. mellea* | 50.97 | | CYP5144NSF5 | 10491 | *A. mellea* |
| CYP5144NSF4 | 9426 | *A. mellea* | 54.35 | | CYP5144NSF4 | 14377.2 | *A. mellea* |
| CYP5144NSF1 | 10753 | *A. mellea* | 73.84 | | CYP5144NSF1 | 5733 | *A. mellea* |
| CYP5144NSF1 | 10801 | *A. mellea* | 76.83 | | CYP5144NSF1 | 5733 | *A. mellea* |
| CYP5144NSF1 | 14062 | *A. mellea* | 58.24 | | CYP5144NSF1 | 5733 | *A. mellea* |
| CYP5144NSF1 | 1044 | *A. mellea* | 66.77 | | CYP5144NSF1 | 5733 | *A. mellea* |
| CYP5144NSF1 | 5382 | *A. mellea* | 65.71 | | CYP5144NSF1 | 5733 | *A. mellea* |
| CYP5144NSF1 | 14064 | *A. mellea* | 68.16 | | CYP5144NSF1 | 5733 | *A. mellea* |
| CYP5144NSF1 | 14063 | *A. mellea* | 72.05 | | CYP5144NSF1 | 5733 | *A. mellea* |
| CYP5144NSF1 | 3062 | *A. mellea* | 69.31 | | CYP5144NSF1 | 5733 | *A. mellea* |
| CYP5348NSF1 | 4316 | *A. mellea* | 45.52 | | CYP5348N1 |  | *P. placenta* |
| CYP5348NSF1 | 3759 | *A. mellea* | 67.55 | | CYP5348NSF1 | 4316 | *A. mellea* |
| CYP620NSF1 | 7995 | *A. mellea* | 56.86 | | CYP5037NSF | 14128 | *A. mellea* |
| CYP620NSF1 | 14128 | *A. mellea* | 42 | | CYP620H6 |  | *A. niger* |
| CYP5037NSF2 | 3761 | *A. mellea* | 47 | | CYP5037B1 |  | *L. edodes* |
| CYP5037NSF3 | 7284 | *A. mellea* | 42.44 | | CYP5037NSF2 | 3761 | *A. mellea* |
| CYP5037NSF4 | 12595 | *A. mellea* | 43 | | CYP5037B3 |  | *P. chrysosporium* |
| CYP5037NSF5 | 12161 | *A. mellea* | 44.55 | | CYP5037NSF4 | 12595 | *A. mellea* |
| CYP5037NSF6 | 3909 | *A. mellea* | 42.82 | | CYP5037NSF4 | 12595 | *A. mellea* |
| CYP5037NSF7 | 7931 | *A. mellea* | 46 | | CYP5037B3 |  | *P. chrysosporium* |
| CYP5037NSF2 | 13551 | *A. mellea* | 81.51 | | CYP5037NSF2 | 3761 | *A. mellea* |
| CYP5037NSF8 | 2217 | *A. mellea* | 45 | | CYP5037B3 |  | *P. chrysosporium* |
| CYP5037NSF8 | 4534 | *A. mellea* | 62.16 | | CYP5037NSF8 | 2217 | *A. mellea* |
| CYP5037B | 11751 | *A. mellea* | 65 | | CYP5037B1 |  | *L. edodes* |
| CYP5037B | 14445 | *A. mellea* | 61.75 | | CYP5037B | 11751 | *A. mellea* |
| CYP5037B | 14446 | *A. mellea* | 57.49 | | CYP5037B | 11751 | *A. mellea* |
| CYP5037B | 3033 | *A. mellea* | 60.38 | | CYP5037B | 14445 | *A. mellea* |
| CYP5037NSF10 | 12856 | *A. mellea* | 48 | | CYP5037B1 |  | *L. edodes* |
| CYP5037NSF11 | 14218 | *A. mellea* | 45 | | CYP5037B3 |  | *P. chrysosporium* |
| CYP5037NSF12 | 11941 | *A. mellea* | 47 | | CYP5037B2 |  | *P. chrysosporium* |
| CYP5037NSF12 | 364 | *A. mellea* | 61.34 | | CYP5037NSF12 | 11941 | *A. mellea* |
| CYP5037 | 13137 | *A. mellea* | 47.26 | | CYP5037NSF2 | 3761 | *A. mellea* |
| CYP5037 | 13132 | *A. mellea* | 50.83 | | CYP5037NSF2 | 13551 | *A. mellea* |
| CYP5037NSF2 | 10705 | *A. mellea* | 85.08 | | CYP5037NSF2 | 13551 | *A. mellea* |
| CYP5037 | 2118 | *A. mellea* | 41.96 | | CYP5037B | 11751 | *A. mellea* |
| CYP5037B | 3386 | *A. mellea* | 61.06 | | CYP5037B | 11751 | *A. mellea* |
| CYP5037B | 14447.1 | *A. mellea* | 68.03 | | CYP5037B | 11751 | *A. mellea* |
| CYP5037B | 14447.2 | *A. mellea* | 89.34 | | CYP5037B | 11751 | *A. mellea* |
| CYP5037B | 11610 | *A. mellea* | 68.67 | | CYP5037B | 11751 | *A. mellea* |
| CYP5037 | 5235 | *A. mellea* | 53.31 | | CYP5037B | 14445 | *A. mellea* |
| CYP5037 | 3509 | *A. mellea* | 53.33 | | CYP5037B | 14446 | *A. mellea* |
| CYP5156NSF | 4613 | *A. mellea* | 50 | | CYP5156A1 |  | *P. chrysosporium* |
| NF1SF1 | 3446 | *A. mellea* | 81.05 | | NF1SF1 | 1944 | *A. mellea* |
| NF1SF1 | 1944 | *A. mellea* | 64.6 | | NF1SF1 | 3445 | *A. mellea* |
| NF1SF1 | 3445 | *A. mellea* | 66.25 | | NF1SF1 | 3446 | *A. mellea* |
| CYP5027NSF | 8161 | *A. mellea* | 47 | | CYP5027A1 |  | *U. maydis* |
| CYP512NSF1 | 10711 | *A. mellea* | 43 | | CYP512E1 |  | *P. chrysosporium* |
| CYP512NSF1 | 13032 | *A. mellea* | 75.37 | | CYP512NSF1 | 10711 | *A. mellea* |
| CYP512NSF1 | 12745 | *A. mellea* | 65.22 | | CYP512NSF1 | 10711 | *A. mellea* |
| CYP512NSF1 | 7410 | *A. mellea* | 56.95 | | CYP512NSF1 | 10711 | *A. mellea* |
| CYP512NSF1 | 14197 | *A. mellea* | 67.77 | | CYP512NSF1 | 7410 | *A. mellea* |
| CYP512NSF1 | 5752 | *A. mellea* | 58.72 | | CYP512NSF1 | 10711 | *A. mellea* |
| CYP512NSF1 | 13186 | *A. mellea* | 61.83 | | CYP512NSF1 | 10711 | *A. mellea* |
| CYP512NSF1 | 3980 | *A. mellea* | 57.24 | | CYP512NSF1 | 10711 | *A. mellea* |
| CYP512NSF1 | 6320 | *A. mellea* | 72.82 | | CYP512NSF1 | 13186 | *A. mellea* |
| CYP512NSF1 | 9479 | *A. mellea* | 75.93 | | CYP512NSF1 | 12745 | *A. mellea* |
| CYP512NSF1 | 7409 | *A. mellea* | 83.27 | | CYP512NSF1 | 12745 | *A. mellea* |
| CYP512NSF1 | 9478 | *A. mellea* | 74.05 | | CYP512NSF1 | 7410 | *A. mellea* |
| CYP512NSF1 | 576 | *A. mellea* | 62.81 | | CYP512NSF1 | 10711 | *A. mellea* |
| CYP512NSF1 | 577 | *A. mellea* | 64.18 | | CYP512NSF1 | 10711 | *A. mellea* |
| CYP6005H | 417 | *A. mellea* | 63.8 | | CYP6005H5 | 161482 | *F. mediterranea* |
| CYP6005H | 3466 | *A. mellea* | 78.61 | | CYP6005H | 417 | *A. mellea* |
| CYP51F | 9976 | *A. mellea* | 60 | | CYP51F1 |  | *P. chrysosporium* |
| CYP51F | 8571 | *A. mellea* | 65 | | CYP51F1 |  | *P. chrysosporium* |
| CYP61A | 4382 | *A. mellea* | 72 | | CYP61A1 |  | *P. chrysosporium* |
| CYP5343NSF1 | 482 | *A. mellea* | 40 | | CYP5343A1 |  | *P. placenta* |
| CYP5065NSF1 | 4550 | *A. mellea* | 43.12 | | CYP5065B1 |  | *Ganoderma sp.* |
| CYP5348NSF1 | 13954 | *A. mellea* | 44.62 | | CYP5348N2v2 |  | *P. placenta* |
| CYP5144NSF1 | 5298 | *A. mellea* | 45.39 | | CYP5144M1 |  | *P. placenta* |
| CYP5035NSF1 | 12713 | *A. mellea* | 48.09 | | CYP5035U4 |  | *B. adusta* |
| NF8SF1 | 7534 | *A. mellea* | 45.27 | | NF8SF2 | 7532 | *A. mellea* |
| CYP5340D1 | 12692 | *A. mellea* | 48.05 | | Named by Prof David R Nelson | | |
| CYP5340 | 13604 | *A. mellea* | 55.34 | | CYP5340D1 | 12692 | *A. mellea* |
| CYP5340 | 7290 | *A. mellea* | 49.32 | | CYP5340D1 | 12692 | *A. mellea* |
| CYP5366B1 | 11549 | *A. mellea* |  | | Named by Prof David R Nelson | | |
| CYP5366B | 5700 | *A. mellea* |  | | CYP5366B1 | 11549 | *A. mellea* |
| ***Melampsora laricis-populina*** | | | | | | | |
|  |  |  | |  | **Reference P450s** | | |
| **P450 name** | **Protein ID** | **Species** | | **Identity %** | **P450 name** | **Protein ID** | **Species** |
| CYP5233NSF1 | 109700 | *M. laricis-populina* | | 45 | CYP5233A1 |  | *P. graminis* |
| CYP5233NSF1 | 110178 | *M. laricis-populina* | | 85.33 | CYP5233NSF1 | 109700 | *M. laricis-populina* |
| CYP5233NSF1 | 117454 | *M. laricis-populina* | | 68.73 | CYP5233NSF1 | 109700 | *M. laricis-populina* |
| CYP5233NSF1 | 123957 | *M. laricis-populina* | | 84.17 | CYP5233NSF1 | 109700 | *M. laricis-populina* |
| CYP5233NSF1 | 86535 | *M. laricis-populina* | | 84.5 | CYP5233NSF1 | 109700 | *M. laricis-populina* |
| CYP5233NSF1 | 123958 | *M. laricis-populina* | | 62.23 | CYP5233NSF1 | 109700 | *M. laricis-populina* |
| CYP5233NSF1 | 66135 | *M. laricis-populina* | | 85.33 | CYP5233NSF1 | 109700 | *M. laricis-populina* |
| CYP5233NSF2 | 109660 | *M. laricis-populina* | | 43 | CYP5233A1 |  | *P. graminis* |
| CYP5221NSF1 | 115340 | *M. laricis-populina* | | 49 | CYP5221C1 |  | *P. graminis* |
| CYP5221C | 89437 | *M. laricis-populina* | | 57 | CYP5221C1 |  | *P. graminis* |
| CYP5221C | 90397 | *M. laricis-populina* | | 56 | CYP5221C1 |  | *P. graminis* |
| CYP5230NSF | 59429 | *M. laricis-populina* | | 50 | CYP5230A1 |  | *P. graminis* |
| CYP5139J | 93703 | *M. laricis-populina* | | 57.68 | CYP5139J1 | 73879 | *M. laricis-populina* |
| CYP5139J1 | 73879 | *M. laricis-populina* | |  | Named by Prof David R Nelson | | |
| CYP5139J1 | 73879 | *M. laricis-populina* | | 78.25 | NF11SF1 | 76380 | *M. laricis-populina* |
| CYP5139J1 | 73879 | *M. laricis-populina* | | 40 | CYP5139C1 |  | *S. roseus* |
| CYP5398A1 | 118362 | *M. laricis-populina* | |  | Named by Prof David R Nelson | | |
| CYP53B3 | 36399 | *M. laricis-populina* | | 60 | CYP53B |  | *P. graminis* |
| CYP67A | 123517 | *M. laricis-populina* | | 57 | CYP67A2 |  | *P. graminis* |
| CYP67A | 46649 | *M. laricis-populina* | | 57 | CYP67A2 |  | *P. graminis* |
| CYP5232NSF | 118500 | *M. laricis-populina* | | 42 | CYP5232A1 |  | *P. graminis* |
| CYP5396A1 | 94377 | *M. laricis-populina* | |  | Named by Prof David R Nelson | | |
| CYP5395A1 | 123422 | *M. laricis-populina* | | 21 | Named by Prof David R Nelson | | |
| CYP5397A1 | 87182 | *M. laricis-populina* | |  | Named by Prof David R Nelson | | |
| CYP5152NSF | 123434 | *M. laricis-populina* | | 54 | CYP5152B1 |  | *P. graminis* |
| CYP5231A | 47825 | *M. laricis-populina* | | 58 | CYP5231A1 |  | *P. graminis* |
| CYP51F1 | 46108 | *M. laricis-populina* | | 67 | CYP51F1 |  | *P. graminis* |
| CYP5139J | 92310 | *M. laricis-populina* | | 72.54 | CYP5139J1 | 73879 | *M. laricis-populina* |
| ***Melampsora lini* CH5** | | | | | | | |
|  |  |  | |  | **Reference P450s** | | |
| **P450 name** | **Protein ID** | **Species** | | **Identity %** | **P450 name** | **Protein ID** | **Species** |
| CYP53B | 209017 | *Melampsora lini* | | 90 | CYP53B3 | 36399 | *M. laricis-populina* |
| CYP5221NSF1 | 201316 | *Melampsora lini* | | 86 | CYP5221NSF1 | 115340 | *M. laricis-populina* |
| CYP5233NSF1 | 197130 | *Melampsora lini* | | 79.54 | CYP5233NSF1 | 109700 | *M. laricis-populina* |
| CYP5139J | 199058 | *Melampsora lini* | | 66.73 | CYP5139J1 | 73879 | *M. laricis-populina* |
| CYP5398A | 199047 | *Melampsora lini* | | 90.55 | CYP5398A1 | 118362 | *M. laricis-populina* |
| CYP5397A | 209632 | *Melampsora lini* | | 73.97 | CYP5397A1 | 87182 | *M. laricis-populina* |
| CYP5399A1 | 201482 | *Melampsora lini* | | Named by Prof David R Nelson | | | |
| CYP5233NSF | 204519 | *Melampsora lini* | | 54.46 | CYP5233NSF2 | 109660 | *M. laricis-populina* |
| CYP5152NSF | 196934 | *Melampsora lini* | | 87.78 | CYP5152NSF | 123434 | *M. laricis-populina* |
| CYP5396NSF | 205583 | *Melampsora lini* | | 51.61 | CYP5396A1 | 94377 | *M. laricis-populina* |
| CYP5231A | 206908 | *Melampsora lini* | | 76.57 | CYP5231A | 47825 | *M. laricis-populina* |
| CYP5230NSF | 198975 | *Melampsora lini* | | 93.62 | CYP5230NSF | 59429 | *M. laricis-populina* |
| CYP5221NSF1 | 201310 | *Melampsora lini* | | 91.06 | CYP5221NSF1 | 115340 | *M. laricis-populina* |
| CYP5233NSF2 | 201440 | *Melampsora lini* | | 82.04 | CYP5233NSF2 | 109660 | *M. laricis-populina* |
| CYP67A | 197037 | *Melampsora lini* | | 90.78 | CYP67A | 123517 | *M. laricis-populina* |
| CYP5139J | 199056 | *Melampsora lini* | | 74.21 | CYP5139J | 93703 | *M. laricis-populina* |
| CYP5139J | 196234 | *Melampsora lini* | | 69.78 | CYP5139J1 | 73879 | *M. laricis-populina* |
| CYP5396A | 201038 | *Melampsora lini* | | 92.03 | CYP5396A1 | 94377 | *M. laricis-populina* |
| CYP5139J | 196509 | *Melampsora lini* | | 85.9 | CYP5139J1 | 73879 | *M. laricis-populina* |
| CYP5232NSF | 199486 | *Melampsora lini* | | 90.56 | CYP5232NSF | 118500 | *M. laricis-populina* |
| CYP5221C | 203927 | *Melampsora lini* | | 74.41 | CYP5221C | 90397 | *M. laricis-populina* |
| ***Mixia osmundae* IAM 14324 v1.0** | | | | | | | |
|  |  |  | |  | **Reference P450s** | | |
| **P450 name** | **Protein ID** | **Species** | | **Identity %** | **P450 name** | **Protein ID** | **Species** |
| CYP5221E1 | 54045 | *Mixia osmundae* | | 43 | CYP5221A1 |  | *S. roseus* |
| CYP5141M1 | 86923 | *Mixia osmundae* | | Named by Prof David R Nelson | | | |
| CYP5662A1 | 102957 | *Mixia osmundae* | | Named by Prof David R Nelson | | | |
| CYP53B5 | 46843 | *Mixia osmundae* | | 53 | CYP53B1 |  | *R. minuta* |
| CYP5663A1 | 53881 | *Mixia osmundae* | | Named by Prof David R Nelson | | | |
| CYP5664A1 | 97149 | *Mixia osmundae* | | Named by Prof David R Nelson | | | |
| CYP5665A1 | 610488 | *Mixia osmundae* | | Named by Prof David R Nelson | | | |
| CYP51F | 15559 | *Mixia osmundae* | | 56 | CYP51F1 |  | *P. graminis* |
| CYP5666A1 | 91768 | *Mixia osmundae* | | Named by Prof David R Nelson | | | |
| CYP5667A1 | 46499 | *Mixia osmundae* | | Named by Prof David R Nelson | | | |
| CYP5668A1 | 94521 | *Mixia osmundae* | | Named by Prof David R Nelson | | | |
| CYP61A | 93604 | *Mixia osmundae* | | 64 | CYP61A1 |  | *U. maydis* |
| CYP5139S1 | 97442 | *Mixia osmundae* | | 41 | CYP5139C1 |  | *S. roseus* |
| CYP5669A1 | 617015 | *Mixia osmundae* | | Named by Prof David R Nelson | | | |
| ***Rhodosporidium toruloides* NP11** | | | | | | | |
|  |  |  | |  | **Reference P450s** | | |
| **P450 name** | **Protein ID** | **Species** | | **Identity %** | **P450 name** | **Protein ID** | **Species** |
| CYP5660A1 | 2495 | *R. toruloides* | | Named by Prof David R Nelson | | | |
| CYP53B | 6808 | *R. toruloides* | | 73 | CYP53B2 |  | *S. roseus* |
| CYP5231NSF | 4236 | *R. toruloides* | | 55 | CYP5231A1 | 37166 | *P. graminis* |
| CYP5093E1 | 4946 | *R. toruloides* | | Named by Prof David R Nelson | | | |
| CYP51 | 3850 | *R. toruloides* | | 84 | CYP51F1 |  | *S. roseus* |
| CYP5139NSF | 381 | *R. toruloides* | | 49 | CYP5139C1 |  | *S. roseus* |
| CYP5221A | 4126 | *R. toruloides* | | 64 | CYP5221A1 |  | *S. roseus* |
| CYP5661A1 | 4181 | *R. toruloides* | | Named by Prof David R Nelson | | | |
| CYP5065A | 6289 | *R. toruloides* | | 65 | CYP5065A2 |  | *N. haematococca* |
| CYP5139C1 | 6360 | *R. toruloides* | | 59 | CYP5139C1 |  | *S. roseus* |
| CYP5660A2 | 7079 | *R. toruloides* | | Named by Prof David R Nelson | | | |
| CYP61A | 1251 | *R. toruloides* | | 78 | CYP61A1 |  |  |
| CYP5222B1 | 164 | *R. toruloides* | | Named by Prof David R Nelson | | | |
| CYP6009A1 | 4419 | *R. toruloides* | | Named by Prof David R Nelson | | | |
| CYP6010A1 | 7658 | *R. toruloides* | | Named by Prof David R Nelson | | | |
| ***Pseudozyma antarctica* T-34** | | | | | | | |
|  |  |  | | **Reference P450s** | | | |
| **P450 name** | **Protein ID** | **Species** | | **Identity %** | **P450 name** | **Protein ID** | **Species** |
| CYP5028A | 79482 | *P. antarctica* | | 82 |  |  | *U. maydis* |
| CYP53C | 80655 | *P. antarctica* | | 87 |  |  | *U. maydis* |
| CYP5029NSF | 80604 | *P. antarctica* | | 48 |  |  | *U. maydis* |
| CYP5640C1 | 81785 | *P. antarctica* | | 53.66 |  |  | *P. hubeiensis* SY62 |
| CYP540NSF | 82698 | *P. antarctica* | | 45 |  |  | *A. terreus* |
| CYP504C | 81921 | *P. antarctica* | | 82 |  |  | *U. maydis* |
| CYP5034A | 80847 | *P. antarctica* | | 81 |  |  | *U. maydis* |
| CYP51F1 | 81702 | *P. antarctica* | | 82 |  |  | *U. maydis* |
| CYP5033A | 84372 | *P. antarctica* | | 66 |  |  | *U. maydis* |
| CYP5031A | 83195 | *P. antarctica* | | 68 |  |  | *U. maydis* |
| CYP5032A | 84654 | *P. antarctica* | | 80 |  |  | *U. maydis* |
| CYP5029A | 84716 | *P. antarctica* | | 73 |  |  | *U. maydis* |
| CYP5642A1 | 80414 | *P. antarctica* | | Named by Prof David R Nelson | | | |
| CYP5026A | 84093 | *P. antarctica* | | 83 |  |  | *U. maydis* |
| CYP5026B | 84242 | *P. antarctica* | | 87 |  |  | *U. maydis* |
| **CYP5636A2** | **79618** | *P. antarctica* | | **71** |  |  | ***S. reilianum* SRZ2** |
| CYP5638A1 | 82876 | *P. antarctica* | | Named by Prof David R Nelson | | | |
| CYP61A | 80991 | *P. antarctica* | | 88 | CYP61A1 |  | *U. maydis* |
| ***Pseudozyma hubeiensis* SY62** | | | | | | | |
|  |  |  | | **Reference P450s** | | | |
| **P450 name** | **Protein ID** | **Species** | | **Identity %** | **P450 name** | **Protein ID** | **Species** |
| CYP5029NSF | 772 | *P. hubeiensis* | | 46 | CYP5029A1 |  | *U. maydis* |
| CYP5032A | 1537 | *P. hubeiensis* | | 90 | CYP5032A1 |  | *U. maydis* |
| CYP683NSF | 2027 | *P. hubeiensis* | | 47 | CYP683A1 |  | *A. nidulans* |
| CYP5033A | 3537 | *P. hubeiensis* | | 76 | CYP5033A1 |  | *U. maydis* |
| CYP5030A | 4958 | *P. hubeiensis* | | 83 | CYP5030A1 |  | *U. maydis* |
| CYP53C | 848 | *P. hubeiensis* | | 90 | CYP53C1 |  | *U. maydis* |
| CYP5028A1 | 1369 | *P. hubeiensis* | | 85 | CYP5028A1 |  | *U. maydis* |
| CYP5034A1 | 3896 | *P. hubeiensis* | | 91 | CYP5034A1 |  | *U. maydis* |
| CYP5025A1 | 4953 | *P. hubeiensis* | | 86 | CYP5025A1 |  | *U. maydis* |
| CYP5031A | 5322 | *P. hubeiensis* | | 82 | CYP5031A1 |  | *U. maydis* |
| CYP51F1 | 6399 | *P. hubeiensis* | | 84 | CYP51F1 |  | *U. maydis* |
| CYP5029A | 1629 | *P. hubeiensis* | | 82 | CYP5029A1 |  | *U. maydis* |
| CYP61A | 2447 | *P. hubeiensis* | | 87 | CYP61A1 |  | *U. maydis* |
| CYP504D | 2599 | *P. hubeiensis* | | 62 | CYP504D1 |  | *U. maydis* |
| CYP5026A | 3115 | *P. hubeiensis* | | 85 | CYP5026A1 |  | *U. maydis* |
| CYP5026B | 3679 | *P. hubeiensis* | | 91 | CYP5026B1 |  | *U. maydis* |
| CYP5642A3 | 560 | *P. hubeiensis* | | 72.42 | CYP5642A1 |  | *P. antarctica* |
| CYP5640A1 | 5230 | *P. hubeiensis* | | Named by Prof David R Nelson | | | |
| ***Sporisorium reilianum* SRZ2** | | | | | | | |
|  |  |  | | **Reference P450s** | | | |
| **P450 name** | **Protein ID** | **Species** | | **Identity %** | **P450 name** | **Protein ID** | **Species** |
| CYP53C | 31 | *S. reilianum* | | 90 | CYP53C1 |  | *U. maydis* |
| CYP504C | 3466 | *S. reilianum* | | 79 | CYP504C1 |  | *U. maydis* |
| CYP5028A1 | 5335 | *S. reilianum* | | 83 | CYP5028A1 |  | *U. maydis* |
| CYP5642A2 | 6580 | *S. reilianum* | | 71.24 | CYP5642A1 |  | *P. antarctica* |
| CYP51F | 914 | *S. reilianum* | | 84 | CYP51F1 |  | *U. maydis* |
| CYP5640B1 | 1815 | *S. reilianum* | | 48.16 | CYP5640A1 |  | *P. hubeiensis* |
| CYP5031A1 | 1427 | *S. reilianum* | | 78 | CYP5031A1 |  | *U. maydis* |
| CYP5032A | 2211 | *S. reilianum* | | 81 | CYP5032A1 |  | *U. maydis* |
| CYP5029A | 2289 | *S. reilianum* | | 72 | CYP5029A1 |  | *U. maydis* |
| CYP5033A | 4848 | *S. reilianum* | | 74 | CYP5033A1 |  | *U. maydis* |
| **CYP5636A1** | **5185** | *S. reilianum* | | Named by Prof David R Nelson | | | |
| CYP5026A | 4570 | *S. reilianum* | | 83 | CYP5026A1 |  | *U. maydis* |
| CYP5026B | 4719 | *S. reilianum* | | 84 | CYP5026B1 |  | *U. maydis* |
| CYP61A | 390 | *S. reilianum* | | 90 | CYP61A1 |  | *U. maydis* |
| CYP5034A | 234 | *S. reilianum* | | 82 | CYP5034A1 |  | *U. maydis* |
| CYP6007A1 | 1629 | *S. reilianum* | | Named by Prof David R Nelson | | | |
| ***Tilletiaria anomala* UBC 951 v1.0** | | | | | | | |
|  |  |  | | **Reference P450s** | | | |
| **P450 name** | **Protein ID** | **Species** | | **Identity %** | **P450 name** | **Protein ID** | **Species** |
| CYP5028B1 | 276560 | *T. anomala* | | Named by Prof David R Nelson | | | |
| CYP53C | 44504 | *T. anomala* | | 69 | CYP53C1 |  | *U. maydis* |
| CYP5637A1 | 255968 | *T. anomala* | | Named by Prof David R Nelson | | | |
| CYP5031NSF | 155440 | *T. anomala* | | 43 | CYP5031A1 |  | *U. maydis* |
| CYP5031B1 | 253920 | *T. anomala* | | Named by Prof David R Nelson | | | |
| CYP51F | 258404 | *T. anomala* | | 70 | CYP51F1 |  | *M. globosa* |
| CYP5641A1 | 226875 | *T. anomala* | | Named by Prof David R Nelson | | | |
| CYP5639A1 | 8821 | *T. anomala* | | Named by Prof David R Nelson | | | |
| CYP5026B1 | 222991 | *T. anomala* | | 56 | CYP5026B1 |  | *U. maydis* |
| CYP5026NSF | 254100 | *T. anomala* | | 47 | CYP5026A1 |  | *U. maydis* |
| CYP61A1 | 252959 | *T. anomala* | | 78 | CYP61A1 |  | *U. maydis* |
| Short P450s |  |  | |  |  |  |  |
| CYP505 | 230266 | *T. anomala* | | 40 | CYP505D6 |  | *P. chrysosporium* |
| CYP505 | 276164 | *T. anomala* | | 40 | CYP505D6 |  | *P. chrysosporium* |
| CYP505 | 64145 | *T. anomala* | | 47 | CYP505A1 |  | *P. chrysosporium* |
| CYP505 | 230244 | *T. anomala* | | 66 | CYP505A13 |  | *N. fischeri* |
