# Supplementary material for: Genome-Wide Annotation and Comparative Analysis of Cytochrome P450 Monooxygenases in Basidiomycete Biotrophic Plant Pathogens
Source: PLoS One. 2015 Nov 4;10(11):e0142100. doi: 10.1371/journal.pone.0142100 (PMC4633277; doi:10.1371/journal.pone.0142100)
Supplement: S3 Table — (DOCX) [file pone.0142100.s004.docx]

| **CYP name** | ***Armillaria mellea*** | ***Phanerochaete carnosa*** | ***Bjerkandera adusta*** | ***Phlebia brevispora*** | ***P. carnosa*** | ***Agaricus bisporus*** | ***Ganoderma* sp.** | ***Postia placenta*** | ***Serpula lacrymans*** |
| --- | --- | --- | --- | --- | --- | --- | --- | --- | --- |
| CYP51 | 2 | 1 | 1 | 1 | 2 | 1 | 1 | 1 | 1 |
| CYP53 | 8 | 1 | 8 | 1 | 7 | 2 | 1 | 7 | 1 |
| CYP61 | 1 | 1 | 1 | 1 | 1 | 1 | 1 | 1 | 2 |
| CYP63 | 17 | 7 | 5 | 7 | 9 | 6 | 5 | 5 | 7 |
| CYP502 |  | 1 | 3 | 1 | 1 | 1 | 6 | 4 | 2 |
| CYP504 |  |  |  |  |  |  |  |  | 1 |
| CYP505 | 3 | 7 |  | 3 | 4 |  | 3 | 2 |  |
| CYP512 | 14 | 14 | 18 | 6 | 27 | 12 | 18 | 14 | 11 |
| CYP526 |  |  |  |  |  |  |  |  | 1 |
| CYP530 |  |  |  |  |  | 1 |  |  |  |
| CYP537 |  |  |  |  |  |  | 1 | 2 |  |
| CYP548 |  |  |  |  |  |  |  |  | 1 |
| CYP553 |  |  |  |  |  |  |  |  | 1 |
| CYP613 |  |  |  |  |  |  |  |  | 3 |
| CYP620 |  |  |  |  | 1 | 2 |  |  | 4 |
| CYP634 |  |  |  |  |  |  |  |  | 1 |
| CYP642 |  |  |  |  |  |  | 1 |  |  |
| CYP645 |  |  |  |  |  |  |  |  | 1 |
| CYP661 |  |  |  |  |  |  |  |  | 4 |
| CYP5025 |  |  |  |  | 1 |  |  |  | 1 |
| CYP5027 | 1 |  |  | 7 |  |  |  | 9 |  |
| CYP5032 |  |  |  |  |  | 3 |  |  | 1 |
| CYP5035 | 1 | 13 | 6 | 10 | 14 |  | 13 | 3 | 3 |
| CYP5036 |  | 5 | 3 | 5 | 8 |  |  |  | 1 |
| CYP5037 | 30 | 5 | 5 | 10 | 8 | 5 | 5 | 13 | 18 |
| CYP5065 | 1 |  |  | 4 |  | 3 | 1 |  | 2 |
| CYP5068 |  |  |  |  |  | 1 |  |  |  |
| CYP5082 |  |  |  |  |  |  |  |  | 1 |
| CYP5136 | 39 | 5 | 6 | 3 | 8 |  | 9 |  | 5 |
| CYP5137 | 8 | 2 | 1 | 1 | 4 | 1 | 1 | 6 | 6 |
| CYP5138 | 1 | 1 | 1 | 1 | 2 |  | 1 | 1 | 1 |
| CYP5139 | 3 | 1 | 3 | 4 | 11 | 3 | 6 | 8 | 1 |
| CYP5140 |  | 1 | 2 | 1 | 1 | 1 | 1 | 1 | 1 |
| CYP5141 |  | 7 | 8 | 5 | 9 | 7 | 2 | 4 | 5 |
| CYP5142 | 6 | 7 | 5 | 7 | 8 | 1 |  |  | 2 |
| CYP5143 | 2 | 2 | 1 |  | 2 |  |  |  | 2 |
| CYP5144 | 28 | 34 | 67 | 12 | 71 | 43 | 3 | 3 | 29 |
| CYP5145 |  | 3 |  |  | 2 | 1 |  |  | 1 |
| CYP5146 |  | 6 | 7 | 3 | 15 |  |  |  | 2 |
| CYP5147 |  | 6 |  | 2 | 7 |  |  |  |  |
| CYP5148 |  | 2 | 2 | 2 | 7 | 2 | 3 | 1 | 1 |
| CYP5149 |  | 1 |  | 1 | 3 |  |  | 1 |  |
| CYP5150 |  | 7 | 18 | 20 | 10 | 12 | 33 | 23 | 2 |
| CYP5151 |  | 1 | 1 | 2 | 2 | 1 | 1 | 1 | 3 |
| CYP5152 |  | 2 | 3 | 2 | 4 |  | 1 | 2 | 12 |
| CYP5153 |  |  | 1 | 1 | 1 | 1 |  |  |  |
| CYP5154 | 1 | 1 | 2 | 2 | 4 |  |  |  | 5 |
| CYP5155 |  | 1 | 1 |  | 1 |  |  |  |  |
| CYP5156 | 1 | 2 | 1 | 1 | 1 | 1 | 1 | 1 | 5 |
| CYP5157 |  | 1 | 1 |  | 1 |  |  |  | 1 |
| CYP5158 |  | 1 | 3 | 1 | 5 |  | 1 | 2 |  |
| CYP5339 |  |  |  |  |  |  |  | 2 |  |
| CYP5323 |  |  |  | 1 |  |  |  |  |  |
| CYP5340 | 3 |  |  |  |  |  | 3 | 1 |  |
| CYP5341 | 6 |  | 1 |  |  |  | 2 | 3 |  |
| CYP5342 |  |  |  |  |  |  |  | 1 |  |
| CYP5343 | 1 |  |  |  |  |  |  | 1 |  |
| CYP5344 |  |  |  | 43 |  |  |  | 3 |  |
| CYP5346 |  |  |  |  |  |  |  | 1 |  |
| CYP5347 |  |  |  |  |  |  | 1 | 2 |  |
| CYP5348 | 3 |  |  |  |  |  | 4 | 34 |  |
| CYP5349 |  |  |  |  |  |  | 1 | 2 |  |
| CYP5350 |  |  |  | 1 |  |  |  | 11 |  |
| CYP5351 |  |  |  | 4 |  |  | 1 | 1 |  |
| CYP5352 |  |  | 1 |  |  |  |  | 1 |  |
| CYP5353 |  |  |  |  |  |  |  | 1 |  |
| CYP5354 |  |  |  |  |  |  |  | 2 |  |
| CYP5355 |  |  |  |  |  |  |  | 1 |  |
| CYP5356 |  |  |  |  |  |  |  | 1 |  |
| CYP5357 |  |  |  | 1 |  |  | 2 |  |  |
| CYP5358 |  |  |  | 1 |  |  | 3 |  |  |
| CYP5359 |  |  |  |  |  |  | 40 |  |  |
| CYP5360 |  |  |  |  |  |  | 1 |  |  |
| CYP5361 |  |  |  |  |  |  | 2 |  |  |
| CYP5362 |  |  |  |  |  |  | 1 |  |  |
| CYP5363 |  |  |  | 2 |  |  |  |  |  |
| CYP5364 |  |  | 1 |  |  |  | 4 |  |  |
| CYP5365 |  |  | 1 |  |  |  | 1 |  |  |
| CYP5366 | 2 |  |  |  |  |  | 1 |  |  |
| CYP5374 |  |  | 1 |  |  |  |  |  |  |
| CYP5417 | 2 |  |  |  |  |  |  |  |  |
| CYP5424 |  |  | 3 | 2 |  |  |  |  |  |
| CYP5427 |  |  | 3 |  |  |  |  |  |  |
| CYP5428 |  |  | 1 |  |  |  |  |  |  |
| CYP5429 |  |  | 1 |  |  |  |  |  |  |
| CYP5431 | 1 |  |  |  |  |  |  |  |  |
| CYP5433 |  |  |  | 1 |  |  |  |  |  |
| CYP5434 |  |  |  | 23 |  |  |  |  |  |
| CYP5445 |  |  |  |  |  |  |  | 1 |  |
| CYP5622 | 3 |  |  |  |  |  |  |  |  |
| CYP5623 | 1 |  |  |  |  |  |  |  |  |
| CYP6001 |  |  |  |  | 4 |  |  |  |  |
| CYP6005 | 2 |  | 2 | 3 |  |  | 2 |  |  |
| CYP6006 | 1 |  |  |  |  |  |  |  |  |
| NA | 76 |  |  |  |  | 3 |  |  | 7 |
| **P450 count** | **267** | **149** | **199** | **209** | **266** | **115** | **188** | **184** | **159** |
