# Supplementary material for: Genome-Wide Annotation and Comparative Analysis of Cytochrome P450 Monooxygenases in Basidiomycete Biotrophic Plant Pathogens
Source: PLoS One. 2015 Nov 4;10(11):e0142100. doi: 10.1371/journal.pone.0142100 (PMC4633277; doi:10.1371/journal.pone.0142100)
Supplement: S5 Table — The member P450s’ genomic localization such as scaffold/node and the DNA region (start and end) are shown in the table. P450s are presented with their protein IDs. Member P450s that are tandemly duplicated are highlighted in red font. (DOCX) [file pone.0142100.s006.docx]

| **CYP63** | | | |
| --- | --- | --- | --- |
| **Protein ID** | **Node** | **Start** | **End** |
| 8105 | 39657 | 1434 | 3477 |
| 8108 | 39657 | 15854 | 17965 |
| 12596 | 86614 | 37720 | 39865 |
| 1094 | 106361 | 3088 | 7239 |
| 1095 | 106361 | 9017 | 10374 |
| 9356 | 53008 | 2560 | 4981 |
| 1781 | 109806 | 19843 | 22642 |
| 12048 | 81107 | 6046 | 8722 |
| 12997 | 90576 | 24357 | 26951 |
| 13752 | 96158 | 15789 | 18200 |
| 14157 | 98374 | 9686 | 12253 |
| 13816 | 96369 | 16327 | 18977 |
| 12399 | 84156 | 899 | 2980 |
| 10717 | 68096 | 8825 | 10776 |
| 10851 | 69722 | 2545 | 5135 |
| 9537 | 5549 | 8 | 1322 |
| **CYP5037** | | | |
| **Protein ID** | **Node** | **Start** | **End** |
| 3761 | 120750 | 52117 | 54234 |
| 11751 | 78943 | 103859 | 106017 |
| 12595 | 86614 | 34515 | 36161 |
| 14445 | 99928 | 17382 | 19041 |
| 14446 | 99928 | 20352 | 22359 |
| 14447 | 99928 | 24288 | 28593 |
| 13137 | 91423 | 50578 | 52448 |
| 13132 | 91423 | 21517 | 23199 |
| 3033 | 116693 | 16335 | 18371 |
| 7995 | 38449 | 17722 | 19751 |
| 14128 | 98280 | 5687 | 7469 |
| 7284 | 31222 | 3483 | 5791 |
| 13551 | 94663 | 13026 | 15112 |
| 12161 | 81840 | 15687 | 17664 |
| 3909 | 121790 | 2437 | 4512 |
| 7931 | 3771 | 9871 | 11881 |
| 2217 | 112021 | 7042 | 9128 |
| 4534 | 126108 | 40173 | 42292 |
| 5421 | 132303 | 28504 | 30021 |
| 12856 | 89022 | 32457 | 35279 |
| 14218 | 98749 | 32619 | 34383 |
| 11941 | 80362 | 48236 | 50955 |
| 364 | 101536 | 12987 | 14110 |
| 10705 | 67819 | 4874 | 6945 |
| 2118 | 111720 | 13544 | 14641 |
| 3386 | 118278 | 94545 | 96280 |
| 11610 | 77068 | 22530 | 23350 |
| 5235 | 131416 | 170 | 2519 |
| 3509 | 119311 | 245 | 1074 |
| **CYP5136** | | | |
| **Protein ID** | **Node** | **Start** | **End** |
| 6520 | 22931 | 15890 | 19762 |
| 6519 | 22931 | 11067 | 13952 |
| 8159 | 40373 | 1196 | 2948 |
| 2714 | 114763 | 19674 | 22333 |
| 1221 | 106931 | 10301 | 12608 |
| 1226 | 106931 | 30691 | 33076 |
| 1228 | 106931 | 38268 | 40659 |
| 1219 | 106931 | 1937 | 7136 |
| 1234 | 106931 | 63048 | 65884 |
| 995 | 104969 | 20946 | 22877 |
| 994 | 104969 | 17541 | 20001 |
| 993 | 104969 | 15764 | 17189 |
| 4525 | 125942 | 71208 | 72510 |
| 4744 | 128163 | 2359 | 4743 |
| 831 | 103739 | 17605 | 20081 |
| 3988 | 122402 | 9593 | 12807 |
| 9355 | 52969 | 407 | 3379 |
| 11686 | 78057 | 2214 | 4458 |
| 8086 | 3942 | 90 | 1799 |
| 13560 | 94702 | 5696 | 8088 |
| 242 | 100563 | 17937 | 28376 |
| 6997 | 28730 | 2069 | 4344 |
| 13087 | 91136 | 1744 | 4006 |
| 6972 | 28160 | 252 | 2522 |
| 878 | 104172 | 123 | 2241 |
| 6722 | 2562 | 14695 | 15589 |
| 10143 | 62150 | 153 | 969 |
| 11189 | 72579 | 2714 | 3872 |
| 10273 | 63413 | 1 | 874 |
| 12654 | 87051 | 964 | 2567 |
| 6142 | 17890 | 30 | 1744 |
| 8485 | 4421 | 141 | 1223 |
| 9414 | 53537 | 64 | 1865 |
| 6688 | 25117 | 1738 | 3284 |
| 10268 | 63409 | 2 | 1537 |
| 14379 | 99213 | 6884 | 12512 |
| **CYP5137** | | | |
| **Protein ID** | **Node** | **Start** | **End** |
| 3906 | 121788 | 11740 | 14073 |
| 3903 | 121788 | 240 | 5629 |
| 5127 | 130984 | 107349 | 109815 |
| 3998 | 122504 | 25355 | 27679 |
| 7654 | 3481 | 47176 | 50430 |
| 3888 | 121786 | 67962 | 69675 |
| 11881 | 79422 | 26452 | 29285 |
| **CYP5341** | | | |
| **Protein ID** | **Node** | **Start** | **End** |
| 887 | 104191 | 3946 | 8314 |
| 5924 | 14900 | 901 | 3300 |
| 10315 | 63888 | 7133 | 10225 |
| 1196 | 106708 | 15344 | 17612 |
| 1773 | 109781 | 11840 | 14150 |
| 11862 | 79089 | 18315 | 20595 |
| **CYP5233** | | | |
| **Protein ID** | **Scaffold** | **Start** | **End** |
| 109700 | 38 | 543076 | 545139 |
| 117454 | 38 | 527943 | 530403 |
| 109660 | 38 | 332723 | 334816 |
| 110178 | 42 | 306778 | 308904 |
| 123957 | 9 | 55170 | 57742 |
| 86535 | 19 | 112131 | 114219 |
| 123958 | 34 | 683871 | 685963 |
| **CYP5221** | | | |
| **Protein ID** | **Supercontig** | **Start** | **End** |
| 25191 | 2_10 | 150895 | 153774 |
| 30234 | 2_27 | 662342 | 664807 |
| 30233 | 2_27 | 659360 | 662432 |
| 27635 | 2_16 | 36767 | 39777 |
| 27645 | 2_16 | 82423 | 84480 |
| 27632 | 2_16 | 23470 | 27191 |
| 29587 | 2_24 | 168439 | 170346 |
| 29695 | 2_24 | 558693 | 561457 |
|  |  |  |  |
